# Effects of botanical drugs in the treatment of cancer-related fatigue in patients with gastric cancer: A meta-analysis and prediction of potential pharmacological mechanisms

**DOI:** 10.3389/fphar.2022.979504

**Published:** 2022-09-07

**Authors:** Ziming Wang, Zihong Wu, Qiong Xiang, Jingyi Yang, Zhenzhong Xia, Aohan Hao, Enfeng Song, Shasha Mei

**Affiliations:** ^1^ Department of Traditional Chinese Medicine, Renmin Hospital of Wuhan University, Wuhan, China; ^2^ School of Clinical Medicine, Chengdu University of Traditional Chinese Medicine, Chengdu, China

**Keywords:** gastric cancer, cancer-related fatigue, meta-analysis, randomized controlled trial, botanical drugs, network analysis

## Abstract

**Objective:** To systematically review the efficacy and safety of botanical drugs in the treatment of cancer-related fatigue (CRF) caused by gastric cancer (GC) and to determine the underlying pharmacological mechanisms using a network analysis.

**Methods:** Databases such as China National Knowledge Infrastructure (CNKI), SinoMed, Wanfang, Pubmed, Embase, Cochrane Library, and Web of Science were searched for randomized controlled trials (RCTs) from inception to 18 April 2022. Methodological quality assessment was performed using the collaborative tool Cochrane, and data analysis were carried out using RevMan 5.4 and STATA 16 software. The botanical drugs with the highest frequency of use in the included studies was selected. The chemical composition, targets of action, disease targets, and shared targets of these botanical drugs were screened based on network analysis to explore the potential mechanisms of treating CRF in patients with gastric cancer (GC).

**Results:** A total of 13 studies that included 986 patients with gastric CRF met the inclusion criteria. The results showed that botanical drugs could improve the CRF scores of gastric CRF, including the total scores of CRF dichotomous data [Odds Ratio (OR) = 4.22; 95% confidence interval (CI) 1.67–10.68; *p* = 0.002], the total scores of CRF continuous data [Standardized Mean Difference (SMD) = -0.98; 95% CI -1.36 to -0.60; *p* < 0.00001], the affective subscales of Piper Fatigue Scale (PFS) scores [Weighted Mean Difference (MD) = -0.79; 95%CI -0.92 to -0.65; *p* < 0.00001], the sensory subscales of PFS scores (MD = -0.57; 95%CI -0.77 to -0.37; *p* < 0.00001), the behavioral subscales of PFS scores (MD = -1.05; 95% CI -1.29 to -0.82; *p* < 0.00001), Quality of Life Questionnaire Core 30 (QLQ-C30) (MD = 10.53, 95% CI 8.26 to12.80; *p* < 0.00001), and the Karnofsky Performance Status scale (KPS) (MD = 5.18, 95% CI 2.60 to 7.76; *p* < 0.0001). The botanical drugs group had milder adverse effects than the control group. A total of 44 chemical components and 241 potential targets were obtained from the online database and 121 drug targets overlapped with the disease targets of CRF in patients with GC. Moreover, five key active ingredients, namely quercetin, Stigmasterol, luteolin, kaempferol, and isorhamnetin, as well as five key targets including AKT1, TP53, TNF, VEGFA, and CASP3, were screened. In addition, five key signaling pathways, including cancer, Hepatitis B, Prostate cancer, Hepatitis C, and Pancreatic cancer pathways, were obtained through enrichment analysis.

**Conclusion:** The results of the study showed that botanical drugs have positive effects on CRF in patients with GC. However, more well-designed, multicenter, and large sample-sized Randomized Controlled Trials are required to evaluate the effectiveness of botanical drugs on CRF in patients with GC.

## 1 Introduction

Gastric cancer (GC), the fifth most common cancer in the world, resulted in the death of more than 750,000 people in 2020 (Sung et al., 2021), posing a serious threat to human health. Currently, the main treatments for GC are surgical therapy and chemotherapy, which are usually associated with anemia, malnutrition, GI bleeding and so on. A multicenter cooperative study involving over 3,000 cancer patients found that about one-third of GC patients lost more than 10 percent body weight (Dews et al., 1980; Nitenberg and Raynard*.*,2000; Birkmeyer et al.,2002). Cancer-related fatigue (CRF) is a distressing, persistent, and subjective feeling of physical, emotional, or cognitive exhaustion inconsistent with recent physical activity levels, which is associated with cancer or cancer therapy, affecting the quality of life (NCCN, 2018). The *2021 NCCN Clinical Practice Guidelines in Oncology* concludes that physical exercise, yoga, and psychosocial interventions (including mindfulness-based interventions) showed the highest level of evidence (NCCN, 2021) and that there is a lack of effective pharmacological interventions for CRF, which is mostly treated with psychostimulants and antidepressants (Kraft and Bowen., 2005; Bruera et al., 2006). However, one study showed that the effect of methylphenidate on CRF does not differ significantly from placebo (Moraska et al., 2010). To address the limitations of current treatments, new treatments are needed to alleviate CRF in patients with GC.

In traditional Chinese medicine (TCM), fatigue is believed to be caused by the *yin*-*yang* disharmony and the deficiency of *qi* and blood in the human body (Xiong et al., 2021). Botanical drugs have been used in treating fatigue for thousands of years and are still used in East Asia to treat various kinds of fatigue, including CRF, with good results (Lee et al., 2021; Song et al., 2021; Zhang et al., 2021).

An increasing body of evidence suggests that botanical drugs can be used as adjunctive therapy for GC to improve associated symptoms, such as fatigue (Hung et al.,2017; Li, 2020). Although many studies have conducted a meta-analysis on the efficacy and safety of botanical drugs in the treatment of CRF (Su et al.,2014; Zhang et al., 2019), to our knowledge, there is no meta-analysis specifically aimed at CRF due to GC. Therefore, the present study conducted a meta-analysis to evaluate the efficacy of botanical drugs on CRF in patients with GC, with the intention of providing more options for the treatment of CRF due to GC.

Additionally, a network analysis was used to predict the pharmacological mechanism of action of the botanical drugs with the highest frequency of use in the included studies, with the aims of exploring its effects on patients with CRF due to GC, analyzing its active ingredients and targets, and clarifying their relationships with CRF of GC.

## 2 Materials and methods

### 2.1 Study registration

This systematic review protocol has been registered in PROSPERO (registration number: CRD42022324654, available from https://www.crd.york.ac.uk/PROSPERO/display_record.php?RecordID = 324654).

### 2.2 Search strategy

From inception to 18 April 2022, randomized controlled trials (RCTs) on botanical drugs used for gastric CRF were searched in the following seven electronic databases: China National Knowledge Infrastructure (CNKI), SinoMed, Wanfang, Pubmed, Embase, Cochrane Library, and Web of Science. For a comprehensive search of relevant literature, we checked the reference list of all relevant articles to find other studies and also visited the International Clinical Trial Registry by U.S. National Institutes of Health and Chinese Clinical Trials Registry for information. The following keywords were searched: (Stomach Neoplasms OR Cancer of Stomach OR Stomach Cancers OR Gastric Cancer) AND (Fatigue OR cancer related fatigue OR CRF OR fatigue) AND (Chinese Herbal OR traditional Chinese medicine OR Herbal Medicine). The search strategy is described in detail in the Appendix. Two evaluators independently screened the literature, extracted and crosschecked the data. When both parties fail to form a unified evaluation, a third party was invited to participate in the discussion to help reach a decision.

### 2.3 Study selection

#### 2.3.1 Inclusion criteria

1) Study type: a randomized controlled trial. 2) Participants: Patients with pathologically confirmed GC accompanied by fatigue, regardless of pathological type, cancer stage, severity, age, gender, and race. 3) Intervention and control: Patients in the control group were treated with chemotherapy and conventional symptomatic treatment, while patients in the Intervention group were treated with botanical drugs on the basis of the control group. Botanical drugs were defined as single herbal medicine, Chinese patent medicine, herbal formula prescribed by doctors, and herbal injections extracted from natural medicinal herbs. Regarding botanical drugs, there is no limit on the time of usage, dosage, administration, or treatment time.

### 2.3.2 Exclusion criteria

1) Studies with no description of the diagnostic criteria; 2) Systematic reviews and animal experiments; 3) Unavailable original text; 4) Non-RCTs; 5) Other tumor-induced CRF; 6) Interventions or controls inconsistent with this study; 7) If studies were published repeatedly, the later publications were excluded; 8) Unpublished articles.

### 2.3.3 Outcome indicators

The primary outcome indicators were the CRF overall rating scale, including Brit Fatigue Inventory (BFI) (Mendoza et al., 1999), Piper Fatigue Scale (PFS) (Piper et al., 1998), and Multidimensional Fatigue Inventory (MFI) (Smets et al., 1995). The secondary outcome indicators were the affective, sensory, and behavioral subscales of PFS scores, Quality of Life Questionnaire Core 30 (QLQ-C30) (Aaronson et al., 1993), and activity of daily life (ADL)-specific outcomes such as the Karnofsky Performance Status scale (KPS) (Mor et al., 1984).

### 2.4 Data extraction

The following data were extracted from the included studies: 1) Basic information, including the research topic, first author, journal, and time of publication; 2) Baseline characteristics of the study objects, including sample size, age of the patients, and gender and disease status in each group; 3) The specifics and follow-up time of the interventions; 4) Key elements of the risk of bias evaluation; 5) The concerned outcome indicators and outcome measurement data.

### 2.5 Quality evaluation of the included studies

The risk of bias assessment tool Cochrane was used as a criterion to critically assess seven aspects, including the method of random assignment sequences, whether the personnel performing the assignment strictly carried out the outcome assignment of random numbers, whether the investigators and subjects were blinded, whether there were omissions of outcome indicators, whether positive results in the study were selectively reported, and whether there were other factors that could cause bias, respectively.

### 2.6 Statistical methods

The software Revman 5.4 and STATA 16 were used for the meta-analysis. The odds ratio (OR) was used as the effect size for dichotomous outcomes, and standardized mean difference (SMD) was used as the effect size when the results of different scales were included in the study of continuous variables. When the same scale was applied in the study, weighted mean difference (MD) was used as the effect size, with 95% confidence interval. Q test and *I* ^*2*^ test were employed to explore whether there was heterogeneity among the studies. For example, a *p* > 0.10 and *I* ^*2*^ < 50% indicates relatively good homogeneity among the studies, and the fixed effect model should be selected; however, the random effect model was used, and sensitivity analysis was carried out to find the source of heterogeneity. When necessary, further subgroup analysis was conducted to determine heterogeneity in clinical and methodology. *p < 0.05* was considered a statistically significant difference. STATA 16 was used to exclude each study one by one to determine the source of heterogeneity.

### 2.7 Publication bias

Publication bias was evaluated by funnel plots and calculated by STATA 16 using Begg’s/Egger’s tests.

### 2.8 The prediction of potential mechanisms of action of botanical drugs using network analysis

#### 2.8.1 The effective compounds and prediction of potential targets of botanical drugs

Traditional Chinese Medicine Systems Pharmacology Database and Analysis Platform (TCMSP) database (https://old.tcmsp-e.com/tcmsp.php) was used to highlight the most frequently used effective components of botanical drugs. The screening conditions were then determined according to the pharmacokinetic parameters (ADME), with the oral uptake rate (OB) ≥ 30%, and drug-likeness (DL) ≥ 0.18 (Ru et al., 2014). Effective components of botanical drugs were obtained, and their potential action targets were predicted. In the UniProt database (https://www.uniprot.org/), the conditions were set as “Reviewed” and the study species were set as “human”. Moreover, the Gene Symbol corresponding to the target protein was compared and the names of the targets were converted uniformly.

#### 2.8.2 The construction of “CRF in patients with GC―Target” database and the establishment of “botanical drug ― disease” Venn diagram

“Cancer-related fatigue” and “gastric cancer” were taken as keywords, and the disease targets of GC and cancer-related fatigue were searched on OMIM (https://www.omim.org) and Genecard (https://www.genecards.org/). The targets of the effective compounds of botanical drugs, as well as GC and gastric CRF targets, were collected and intersected through venny 2.1.0 online platform so as to construct the “botanical drug ― disease” Venn diagram.

#### 2.8.3 The construction and analysis of PPI network

The screened drug-disease target proteins were imported into the STRING protein interaction database (https://cn.string-db.org/). The human source was defined to obtain the protein-protein interaction (PPI) network relationship. Simultaneously, the obtained TSV format file was imported into the software, cytoscpe 3.7.2, for in-depth analysis. The network topology analysis plug-in, CytoNCA, processed the target network according to Degree and screened out the key targets and main targets.

#### 2.8.4 GO enrichment analysis and Kyoto Encyclopedia of Genes and Genomes (KEGG) enrichment analysis

The gene was explained and annotated using three dimensions: biology process (BP), molecular function (MF), and Cellular Component (CC), and the pathways associated with the targets were detected by KEGG. The key pathways with humanization as the source and false discovery rate (FDR) lower than *p* < 0.05 were screened from the Metascape database (https://metascape.org/).

## 3 Results

### 3.1 Literature search

**FIGURE 1:**
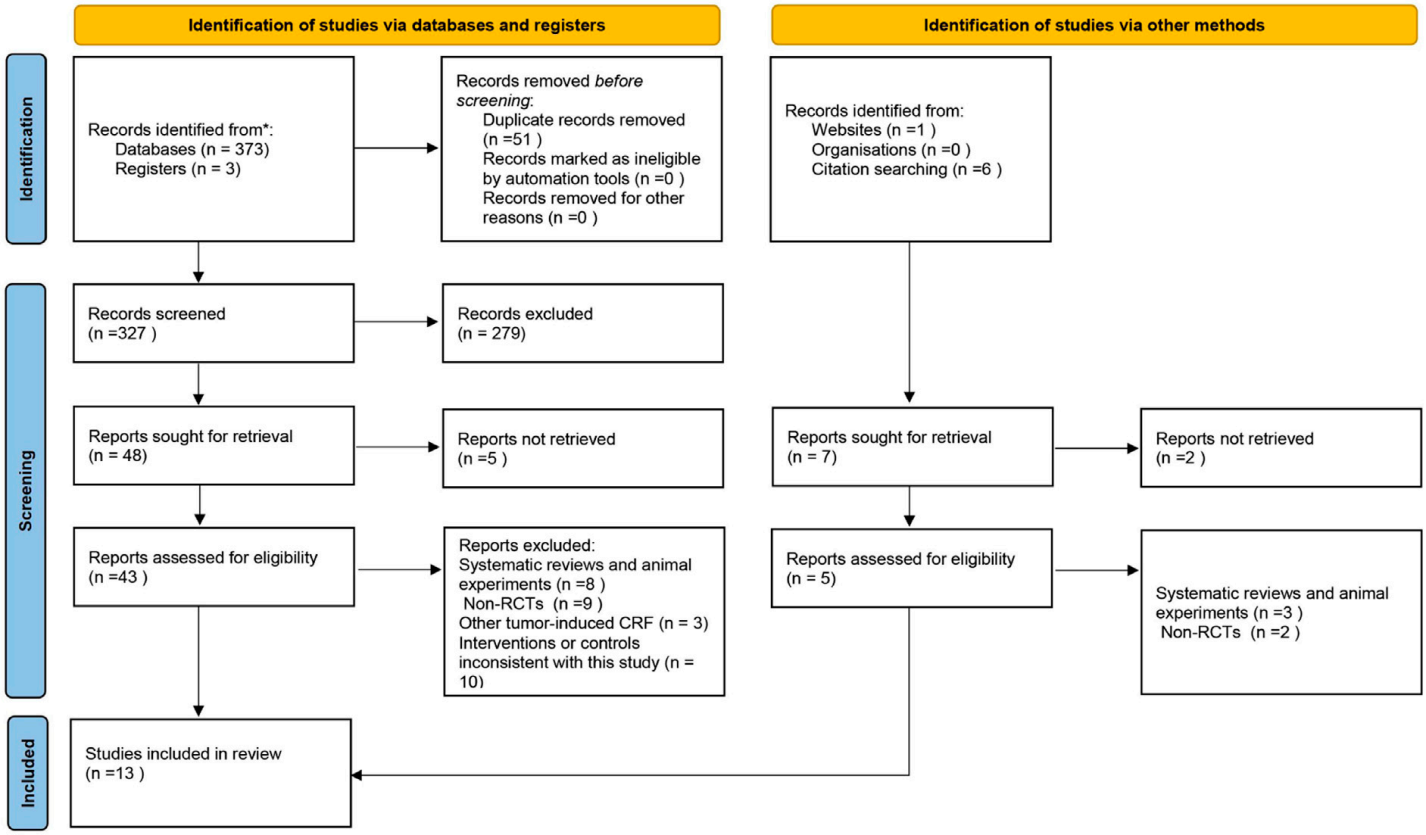
A prisma flow diagram of the literature screening and selection process.

Based on the search strategy, a total of 378 articles were retrieved, which included 51 repeat articles. Finally, 13 of 327 articles were included in the meta-analysis based on the inclusion and exclusion criteria (Fan et al., 2015; Wang et al., 2016; Yong and Jia, 2016.; Hao and Liu, 2018; Wang, 2018; Chen et al., 2019; Gao, 2019; Si et al., 2019; Zhu et al., 2019; Ai and Huang, 2020; Li, 2020; Ma et al., 2020; Gu et al., 2021.). The database search process is summarized in Figure 1.

### 3.2 Basic characteristics of the included studies

The 13 studies were published between 2015 and 2020, including 986 patients. There were 496 cases in the botanical drugs treatment group and 490 cases in the control group, with a treatment duration of 3–12 weeks. The treatment and control groups were comparable in sample size, age, gender, and clinical indications such as CRF score. All the studies reported diagnostic criteria (Fan et al., 2015; Wang et al., 2016; Yong and Jia, 2016.; Hao and Liu, 2018; Wang, 2018; Chen et al., 2019; Gao, 2019; Si et al., 2019; Zhu et al., 2019; Ai and Huang, 2020; Li and Yang, 2020; Ma et al., 2020; Gu et al., 2021), and were published in China (Fan et al., 2015; Wang et al., 2016; Hao and Liu, 2018; Wang, 2018; Chen et al., 2019; Gao, 2019; Si et al., 2019; Zhu et al., 2019; Li, 2020; Ma et al., 2020; Ai and Huang, 2021; Gu et al., 2021; Yong et al., 2021.). One of the studies was a master’s thesis (Gao, 2019), and 12 were journal articles (Fan et al., 2015; Wang et al., 2016; Hao and Liu, 2018; Wang, 2018; Chen et al., 2019; Si et al., 2019; Zhu et al., 2019; Ai and Huang, 2020; Li, 2020; Ma et al., 2020; Gu et al., 2021; Yong et al., 2021). One study reported the affective, sensory, and behavioral subscales of PFS scores but not the total (Ma et al., 2020), and 12 reported the total CRF scores (Fan et al., 2015; Wang et al., 2016; Yong and Jia, 2016; Hao and Liu, 2018; Wang, 2018; Chen et al., 2019; Gao, 2019; Si et al., 2019; Zhu et al., 2019; Li, 2020; Ai and Huang, 2021; Gu et al., 2021). Specifically, three studies used dichotomous data (Fan et al., 2015; Wang et al., 2016; Gao, 2019), and nine employed continuous variable statistics (Hao and Liu, 2018; Wang, 2018; Chen et al., 2019; Si et al., 2019; Zhu et al., 2019; Li, 2020; Ai and Huang, 2021; Gu et al., 2021; Yong et al., 2021), two used the MFI rating scale (Hao and Liu, 2018; Gu et al., 2021), two used the BFI rating scale (Yong et al., 2021; Gao, 2019), nine used the PFS rating scale (Fan et al., 2015; Wang et al., 2016; Wang, 2018; Chen et al., 2019; Si et al., 2019; Zhu et al., 2019; Ai and Huang, 2020; Li, 2020; Ma et al., 2020), and five reported ratings on the affective scores of the CRF continuous data (Wang et al., 2016; Chen et al., 2019; Si et al., 2019; Zhu et al., 2019; Ma et al., 2020). In addition, five studies reported the affective and sensory subscales of PFS scores (Wang et al., 2016; Chen et al., 2019; Si et al., 2019; Zhu et al., 2019; Ma et al., 2020), and four reported the behavioral subscales of PFS scores (Wang et al., 2016; Chen et al., 2019; Zhu et al., 2019; Ma et al., 2020). A total of six studies mentioned adverse effects (Wang et al., 2016; Gao, 2019; Fan et al., 2015; Zhu et al., 2019; Si et al., 2019, Yong et al., 2021). The basic characteristics of the studies are shown in Table 1.

**TABLE 1:** Characteristics of RCTs included in the study.

Study ID	Region	Sample size (T/C)	Age (y)		Gender (M/F)		Intervention		Duration	Outcome
			T	C	T	C	T	C	(weeks)	
Wang, 2018	China	20/20	65.9 ± 10.198	64.4 ± 12.713	12/8	13/7	SOX + Shenqi Fuzheng Injection	SOX	3	a,b,c,d,f
Ma et al., 2020	China	30/30	63.±5.6	62.3 ± 4.3	16/14	17/13	SOX + Xingjian Decoction	SOX	9	a,c,d
Hao and Liu, 2018	China	38/38	64.02 ± 9.15	61.61 ± 10.20	24/14	20/17	Yiqi Yangxue decoction + Basic treatment to symptoms	Basic treatment to symptoms	NA	a
Zhu et al., 2019	China	61/61	62.58 ± 9.33	61.27 ± 11.35	30/31	32/29	FOLFOX4 + Jianpiyishen Formula	FOLFOX4	8	a,b,c,d,e,f
Li, 2020	China	32/30	67.20 ± 6.92	66.74 ± 7.35	22/10	19/11	SOX +Aidi Injection	SOX	12	a,g
Si et al., 2019	China	60/56	58.5 ± 12.5	58.2 ± 11.6	37/23	35/21	Oxaliplatin and capecitabine chemotherapy + Aidi injection	Oxaliplatin and capecitabine chemotherapy	2	a,b,c,e
Chen et al., 2019	China	30/30	52.13 ± 4.17	52.13 ± 4.17	15/15	15/15	FOLFOX4+Bazhen decoction + Shenqi Fuzheng Injection	FOLFOX4	4	a,b,c,d,e
Wang et al., 2016	China	42/42	65-77	66-80	33/9	32/10	Tegio capsule + guipi decoction	Tegio capsule	6	a
Gao, 2019	China	30/30	NA	NA	26/4	23/7	SOX + Shenqi Fuzheng Injection	SOX	6	a,e
Fan et al., 2015	China	30/30	52.73 ± 1. 92	51. 07 ± 1. 85	15/15	14/16	Weifu Formula + FOLFOX6	FOLFOX6	6	a
Ai and Huang, 2021	China	35/36	53.03 ± 2.24	53.85 ± 2.09	18/17	19/17	SOX + Aidi Injection	SOX	12	a
Gu et al., 2021	China	30/30	62.53 ± 5.84	63.07 ± 5.90	21/9	18/12	Buzhong yishen decoction + Basic treatment to symptoms	Basic treatment to symptoms	4	a,e
Yong et al., 2021	China	60/60	55.43 ± 6.94	54.26 ± 6.74	28/20	36/21	ECF + Jianpi Kangai decoction	ECF	9	a,f

T, treatment group; C, control group; NA, not available; M/F, male/female; Outcomes: a, CRF, total score; b, affective subscales of PFS, scores; c, sensory subscales of PFS, scores; d, behavioral subscales of PFS, scores; e, QLQ-C30, score; f, KPS, score; SOX, SOX, chemotherapy regimens; FOLFOX4, FOLFOX4 chemotherapy regimens; FOLFOX6, FOLFOX6 chemotherapy regimens; ECF, ECF, chemotherapy regimens.

### 3.3 Risk of bias

**FIGURE 2:**
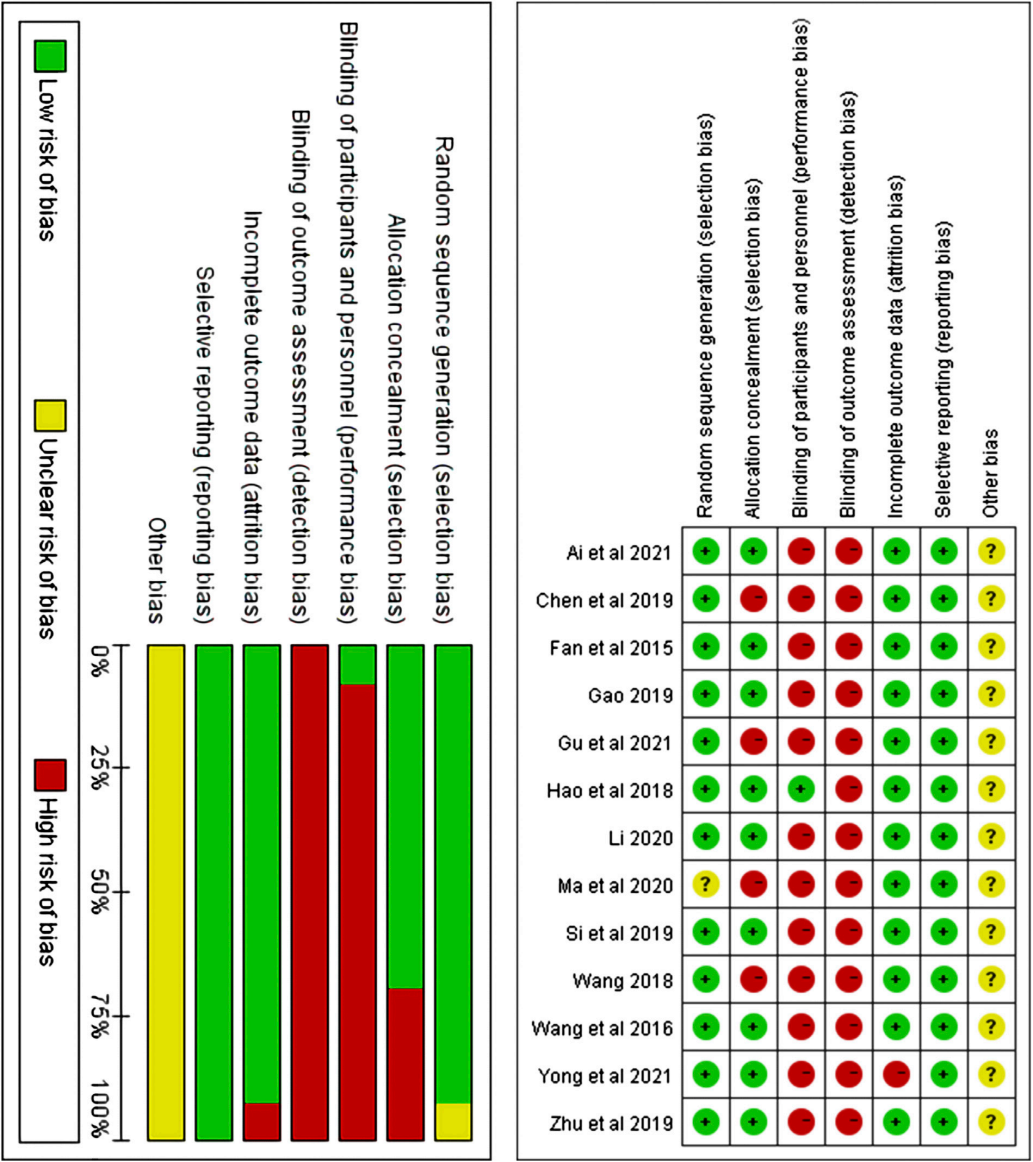
Risk of bias assessment graph for included RCTs and distribution of risk of bias of included RCTs.

A total of 12 studies (Wang et al., 2016; Gao, 2019; Fan et al., 2015; Wang, 2018; Hao and Liu, 2018; Zhu et al., 2019; Li, 2020; Si et al., 2019; Chen et al., 2019; Gu et al.,2021; Yong et al.,2021; Ai and Huang, 2021) described random sequence generation and were, therefore, assessed as having a low risk of bias, whereas, one study (Ma et al., 2020) that did not mention random sequence generation was assessed as having an unclear risk of bias. Four studies (Chen et al., 2019; Gu et al.,2021; Wang, 2018; Ma et al., 2020) lacked a description of allocation concealment; therefore, the domains of allocation concealment was assessed as “high risk”, while the other 9 studies (Wang et al., 2016; Gao, 2019; Fan et al., 2015; Hao and Liu, 2018; Zhu et al., 2019; Li, 2020; Si et al., 2019; Yong et al.,2021; Ai and Huang, 2021) that reported the allocation of hidden descriptions, were assessed as having low risk of bias. A study (Hao and Liu, 2018) described the blinding of participants and personnel and was assessed as “low risk”, while the remaining 12 studies were assessed as “high risk.” Twelve studies (Fan et al., 2015; Wang et al., 2016; Hao and Liu, 2018; Wang, 2018; Chen et al., 2019; Gao, 2019; Si et al., 2019; Zhu et al., 2019; Li and Yang, 2020; Ma et al., 2020; Ai and Huang, 2021; Gu et al., 2021) were assessed as having low risk of bias in the incomplete outcome data domain because there were no reported dropout or withdrawal of participants. However, one study (Yong et al., 2021) was assessed as having a high risk of bias in the incomplete outcome data domain because the documented dropouts or withdrawals were without reason. All the studies mentioned that basic data such as gender and age were comparable between the observation and control groups, and none mentioned selective reporting; hence, the domain of selective reporting was assessed as “low risk.” Although no significant other bias was observed in any of the RCTS, there were many factors leading to other biases; hence, the domains of other biases were assessed as “unclear risk of bias.” The specific evaluation results are shown in Figure 2.

### 3.4 Analysis results

#### 3.4.1 Total CRF dichotomous variable scores

**FIGURE 3:**
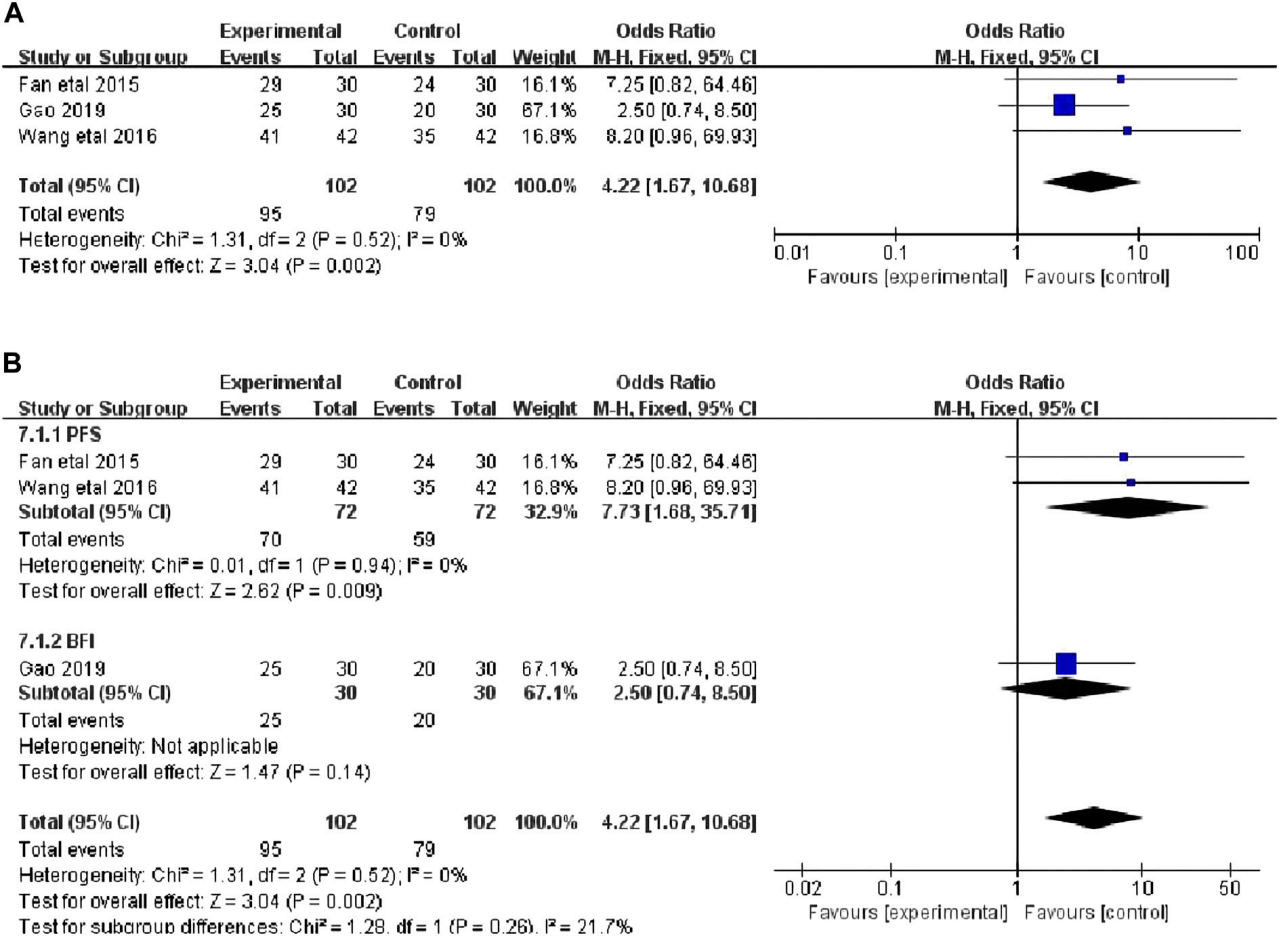
**(A)** The forest plot of total scores of CRF dichotomous data. **(B)** The forest plot of subgroup study of overall rating of CRF dichotomous variables.

Three studies that included 204 patients (102 in the botanical drugs group and 102 in the control group) reported the total CRF dichotomous variable scores (Fan et al., 2015; Wang et al., 2016; Gao, 2019). Heterogeneity test showed *p = 0.52, I* ^*2*^ *= 0%*; therefore, the fixed-effect model was employed. “Events”was the number of patients with no, mild, or moderate fatigue, and“Totals” was the sample size of the intervention or control group. The following categories were highlighted according to the PFS and BFI fatigue Scale: 0-3 score, no fatigue or mild fatigue; 4-6 score, moderate fatigue; 7-10 score, severe fatigue. The results showed that the clinical efficiency in the botanical drugs group was higher than that in the control group (OR = 4.22; 95%CI 1.67 to 10.68; *p* = 0.002, Figure 3A). Since the total score of CRF dichotomous variables consisted of different scales, subgroup analysis was performed. The heterogeneity among the subgroups of PFS and BFI did not differ significantly (PFS: *p* = 0.94, *I* ^*2*^ = 0%), and a fixed-effects model was used. The results of each subgroup study showed that the overall rating of CRF dichotomous variables in the botanical drugs group was higher than that in the control group [PFS: OR = 7.73; 95%CI 1.68 to 35.71; *p* = 0.009, Figure 3B].

#### 3.4.2 The total CRF continuous variable scores

**FIGURE 4:**
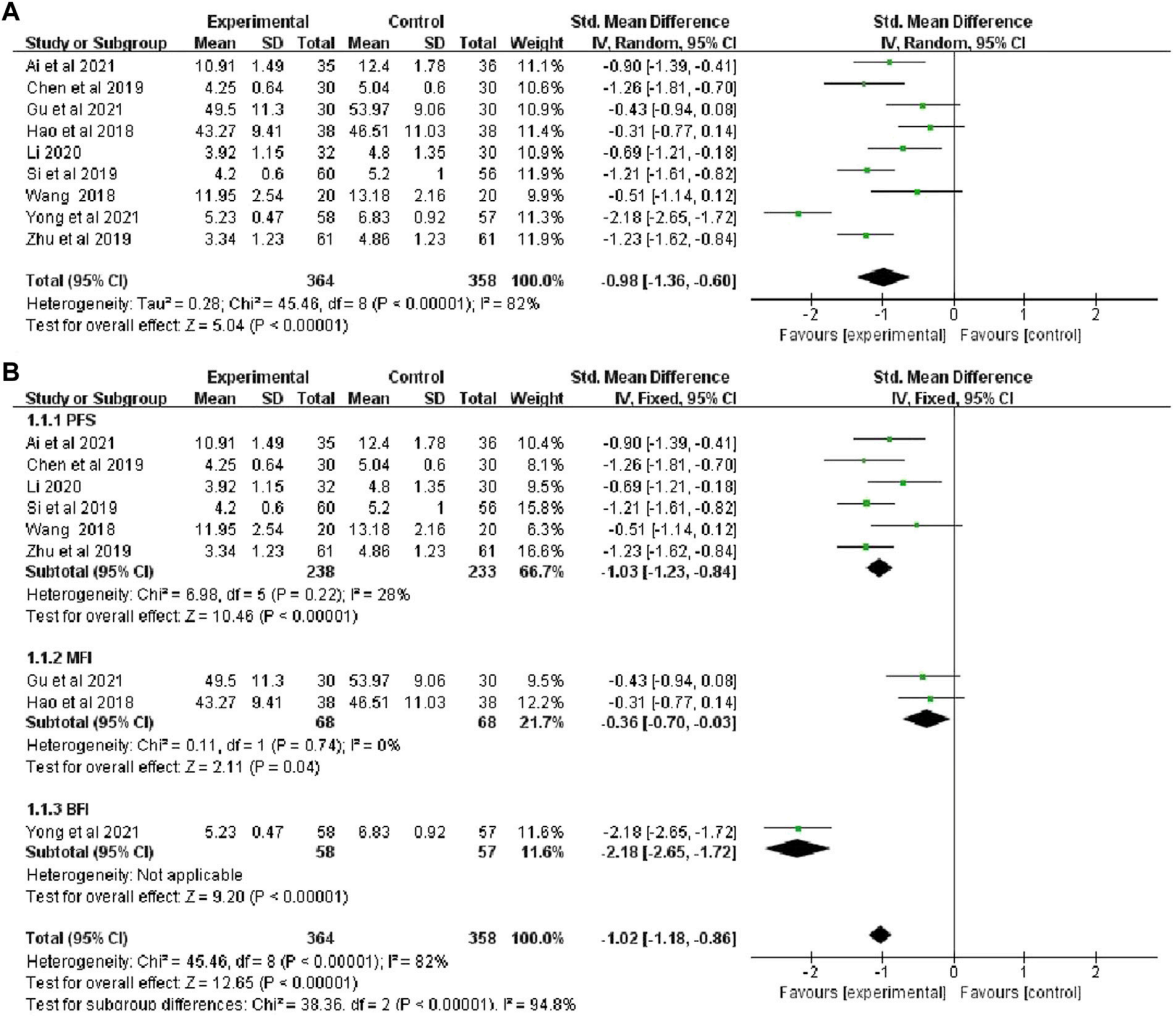
**(A)** The forest plot of total scores of CRF continuous data. **(B)**The forest plot of subgroup study of overall rating of CRF continuous variables.

The total CRF continuous variable scores were reported in nine studies (Ai and Huang, 2021; Gu et al.,2021; Yong et al.,2021; Wang, 2018; Hao and Liu 2018; Zhu et al., 2019; Li, 2020; Si et al., 2019; Chen et al., 2019), that included 722 patients (364 in the botanical drugs group and 358 in the control group). Due to the data heterogeneity among the studies (*p* < 0.00001, *I* ^*2*^ = 82%), the random effect model was employed. The results showed that the total CRF continuous variable scores were better in the botanical drugs group than in the control group (SMD = -0.98, 95%CI -1.36 to -0.60; *p* < 0.00001, Figure 4). Because the heterogeneity of the results was too high, and different CRF rating scale was used in the included study, a subgroup analysis was further performed according to the different rating scales. The results showed that the heterogeneity among the subgroups of PFS, MFI, and BFI was significantly different (PFS:*p* = 0.22, *I* ^*2*^ = 28%; MFI: *p* = 0.74, *I* ^*2*^ = 0%); therefore, a fixed-effects model was used. The results of each subgroup study showed that the overall rating of CRF continuous variables in the botanical drugs group was higher than that in the control group (PFS: SMD = -1.03, 95%CI [-1.23, -0.84], *p* < 0.00001; MFI: SMD = -0.36, 95%CI [-0.70, -0.03], *p* = 0.04, Figure 4).

#### 3.4.3 The affective subscales of PFS scores

**FIGURE 5:**
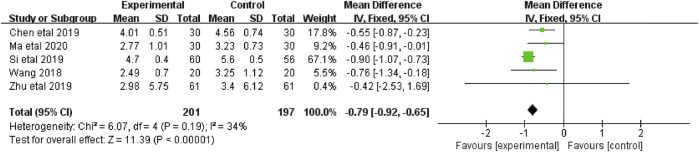
The forest plot of affective subscales of PFS scores.

The affective subscales of PFS scores were reported in five studies (Wang, 2018; Chen et al., 2019; Si et al., 2019; Zhu et al., 2019; Ma et al., 2020), including 398 patients (201 in the botanical drugs group and 197 in the control group). Both groups applied PFS; therefore, the analysis was conducted with MD. Since *I* ^*2*^ < 50% and the data were of low heterogeneity (*p* = 0.19, *I* ^*2*^ = 34%), a fixed-effects model was used. The results demonstrated that the botanical drugs group had better CRF affective scores than the control group (MD = -0.79; 95%CI -0.92 to -0.65; *p* < 0.00001, Figure 5).

#### 3.4.4 The sensory subscales of PFS scores

**FIGURE 6:**
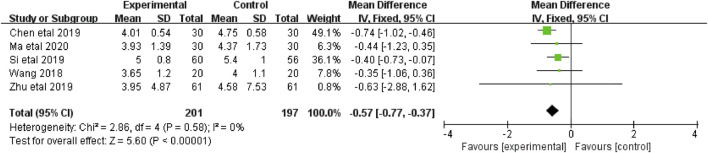
The forest plot of sensory subscales of PFS scores.

The sensory subscales of PFS scores were reported in five studies (Wang, 2018; Chen et al., 2019; Si et al., 2019; Zhu et al., 2019; Ma et al., 2020) that included 398 patients (201 in the botanical drugs group and 197 in the control group). Both groups applied PFS; hence, the analysis was conducted with MD. Since no data heterogeneity (*p* = 0.58; *I* ^*2*^ = 0%) was found, a fixed-effect model was used. The results showed that the CRF sensory scores were better in the botanical drugs group than in the control group (MD = -0.57; 95%CI -0.77 to -0.37; *p* < 0.00001; Figure 6).

#### 3.4.5 The behavioral subscales of PFS scores

**FIGURE 7:**
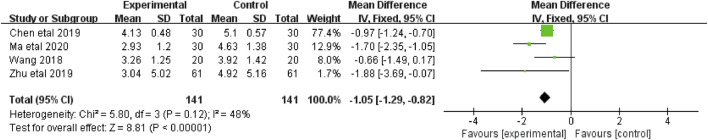
The forest plot of behavioral subscales of PFS scores.

The behavioral subscales of PFS scores were reported in four studies (Wang, 2018; Chen et al., 2019; Zhu et al., 2019; Ma et al., 2020) that included 282 patients (141 in the botanical drugs group and 141 in the control group). Both groups applied PFS; therefore, the analysis was conducted with MD. Since *I* ^*2*^ < 50% and the data were of low heterogeneity (*p* = 0.12, *I* ^*2*^ = 48%), a fixed-effects model was used. The results showed that the botanical drugs group had better CRF behavioral scores than the control (MD = -1.05, 95%CI -1.29 to -0.82; *p* < 0.00001, Figure 7).

#### 3.4.6 QLQ-C30

**FIGURE 8:**
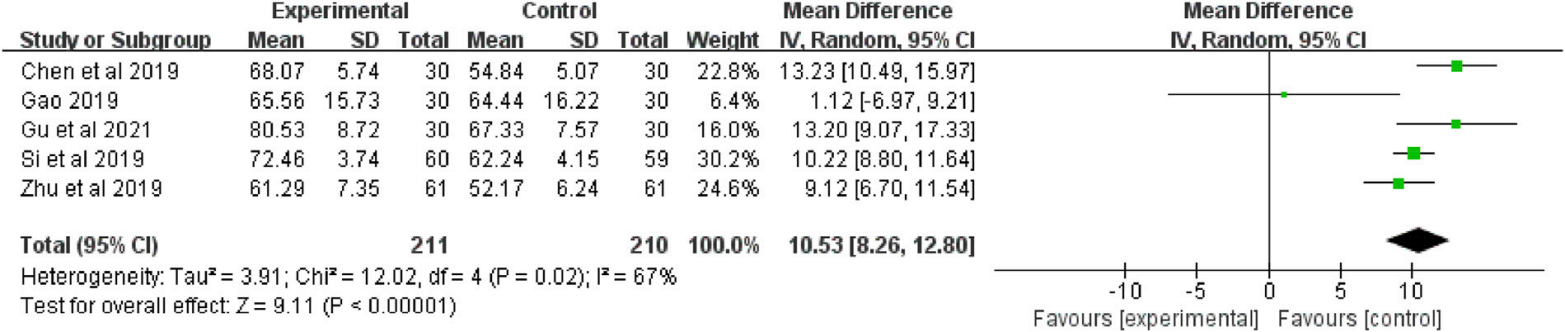
The forest plot of QLQ-C30.

The QLQ-C30 were reported in five studies (Gao 2019; Gu et al., 2021; Zhu et al., 2019; Chen et al., 2019, Si et al., 2019), including 421 patients (211 in the botanical drugs group and 210 in the control group). Both groups applied QLQ-C30; hence, the analysis was conducted with MD. Due to data heterogeneity among the studies (*p* = 0.02, *I* ^*2*^ = 67%), the random effect model was employed. The results showed that the botanical drugs group had better QLQ-C30 scores than the control group (MD = 10.53, 95% CI 8.26 to 12.80; *p* < 0.00001, Figure 8).

#### 3.4.7 KPS

**FIGURE 9:**
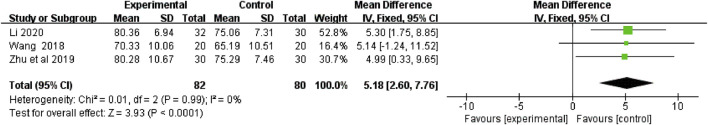
The forest plot of KPS.

The KPS were reported in three studies (Li 2020; 2018; Zhu et al., 2019; Wang 2018), that included 162 patients (82 in the botanical drugs group and 80 in the control group). Both groups applied KPS; therefore, the analysis was conducted with MD. Since no data heterogeneity (*p* = 0.99; *I* ^*2*^ = 0%) was found, a fixed-effect model was used. The results showed that the botanical drugs group had better KPS scores than the control group (MD = 5.18, 95% CI 2.60 to 7.76; *p* < 0.0001, Figure 9).

#### 3.4.8 Adverse reactions

Six studies mentioned adverse reactions (Wang et al., 2016; Gao, 2019; Fan et al., 2015; Zhu et al., 2019; Si et al., 2019, Yong et al., 2021), most of which were myelosuppression and gastrointestinal symptoms caused by chemotherapy. The adverse reactions in the botanical drugs group were milder than those in the control group except for the study by Fan et al., 2015 (Table 2).

**TABLE 2:** Adverse events reported in the included studies.

Study	Adverse events
Wang, 2018	NA
Ma et al., 2020	NA
Hao and Liu, 2018	NA
Zhu et al., 2019	The erythrocytes, hemoglobin, leukocytes and neutrophils of the botanical drug group were better than those of the control group, but there were no statistically significant differences in liver and kidney functions between the two groups.
Li, 2020	NA
Si et al., 2019	The main adverse reactions during treatment in both groups were bone marrow suppression, gastrointestinal reactions, hand-foot syndrome and peripheral neurotoxicity, but the degree of bone marrow suppression in the botanical drug group was significantly less than that in the control group. The differences in other adverse effects were not statistically significant.
Chen et al., 2019	NA
Wang et al., 2016	The gastrointestinal reactions and bone marrow transplantation were less severe in the botanical drug group than in the control group.
Gao, 2019	The incidence of leukopenia, nausea and vomiting, and anorexia in the botanical drug group was significantly lower than that in the control group.
Fan et al., 2015	There was no statistically significant response in the GI tract between the botanical drug and treatment groups.
Yong et al., 2021	The myelosuppression, neurotoxicity and gastrointestinal reaction in the observation group were less serious than the control group.
Ai and Huang, 2021	NA
Gu et al. (2021)	NA

### 3.5 Sensitivity analysis

**FIGURE 10:**
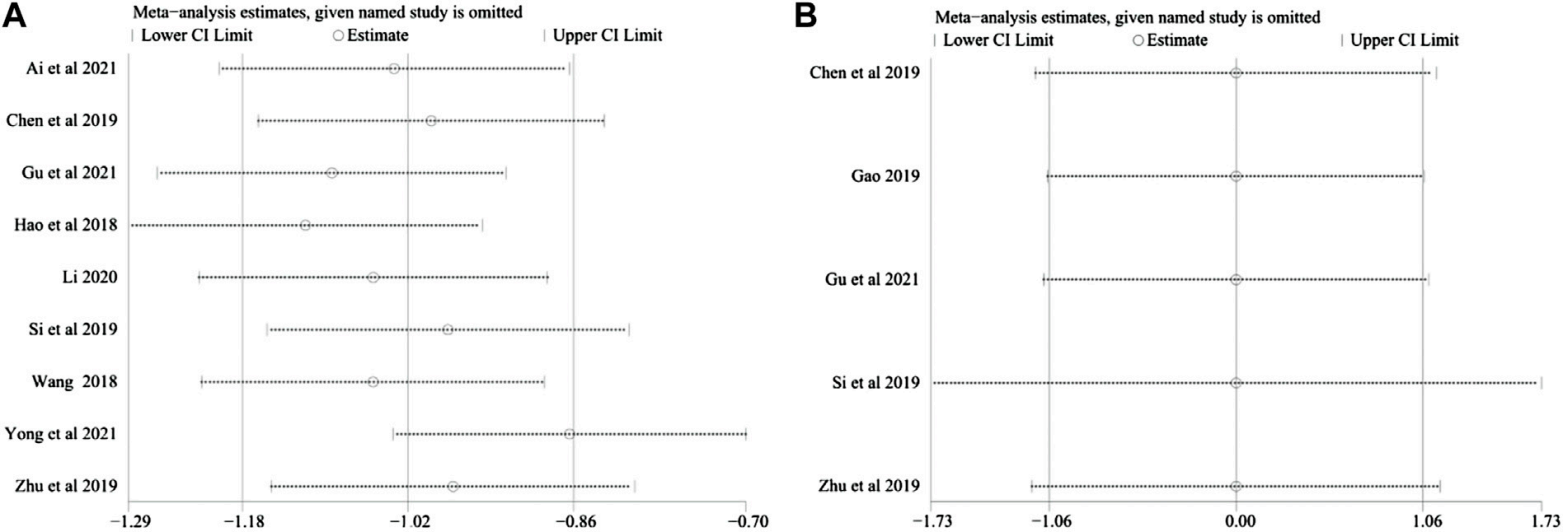
**(A)** Sensitivity analysis plots of total CRF dichotomous variable scores. **(B)** Sensitivity analysis plots of QLQ-C30.

Sensitivity analysis was carried out to investigate the source of the data heterogeneity in the Total CRF continuous variable scores and QLQ-C30 results found among the studies. The sensitivity analysis showed that excluding any study for each outcome did not alter the overall results, indicating that the conclusions were robust (Figure 10).

### 3.6 Publication bias

**FIGURE 11:**
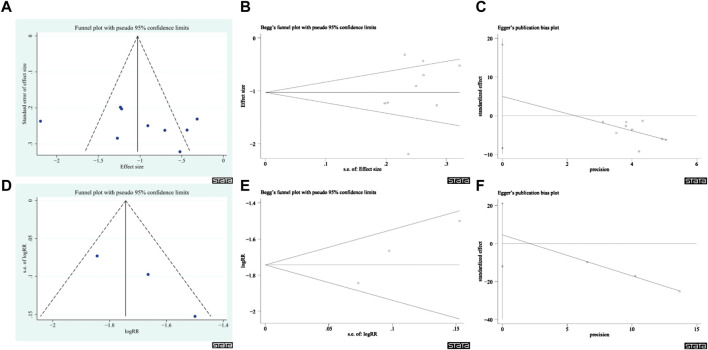
Publication bias plots. **(A)** Funnel plot of CRF Total continuous variable score. **(B)** Begg’s plot of CRF Total continuous variable score. **(C)** Egger’s plot of CRF Total continuous variable score. **(D)** Funnel plot of CRF Total score of Dichotomous data. **(E)** Begg’s plot of CRF Total score of Dichotomous data. **(F)** Egger’s plot of CRF Total score of dichotomous variables.

Publication bias was evaluated based on the results of the total CRF continuous variable scores and the total CRF dichotomous variable scores (Figures 11A–F), and the results showed no publication bias. This conclusion was supported by the results of Egger’s and Begg’s tests (the total CRF continuous variable scores: *z* = 0.94, *p* = 0.348; *t* = 0.88, and *p* = 0.409; the CRF dichotomous variables total score: *z* = 1.04, *p* = 0.296; *t* = 3.48, *p* = 0.178). However, this result should be interpreted with caution due to the small sample size.

### 3.7 Network analysis

The analysis of botanical drugs use in the 13 studies of this evaluation showed that a total of 55 botanical drugs were used, and the top six drugs in terms of frequency of use were Astragalus mongholicus Bunge Fabaceae. (Fabaceae; Astragali Radix), Atractylodes macrocephala Koidz. (Asteraceae; Atractylodis Macrocephalae Rhizoma), Codonopsis pilosula (Franch.) Nannf. (Campanulaceae; Codonopsisradix), *Glycyrrhiza* uralensis Fisch. ex DC. (Fabaceae; Glycyrrhizae radix et rhizome), Poria cocos (Schw.)Wolf. (Polyporaceae; Poria), and Angelica sinensis (Oliv.) Diels (Apiaceae; Angelicae Sinensis Radix), all of which were used more than five times each, with a frequency of use ranging from 46.1% to 92.3% (as shown in Table 3). The remaining botanical drugs were used less than four times each. Consequently, a network analysis of the top six botanical druga in terms of frequency of use was conducted to predict their potential mechanisms for treating gastric CRF.

**TABLE 3:** The most commonly used ingredients in the 13 studies.

Chinese name	Pharmaceutical name	Species	Family	N/13 (%)
Huangqi	*Astragali radix*	*Astragalus mongholicus Bunge*	Fabaceae	12 (92.3%)
Baizhu	*Atractylodes macrocephalae rhizoma*	*Atractylodes macrocephala Koidz*	Asteraceae	8 (61.5%)
Dangshen	*Codonopsisradix*	*Codonopsis pilosula* (Franch.) Nannf.	Campanula-ceae	8 (61.5%)
Gancao	Glycyrrhizae radix et rhizoma	*Glycyrrhiza uralensis Fisch. ex DC.*	Fabaceae	8 (61.5%)
Fuling	Poria	*Poria cocos* (*Schw*.)*Wolf.*	Polyporaceae	7 (53.8%)
Danggui	Angelicae sinensis radix	*Angelica sinensis (Oliv.) Diels*	Apiaceae	6 (46.1%)

#### 3.7.1 The prediction of the effective compounds and potential targets of the botanical drugs

**FIGURE 12:**
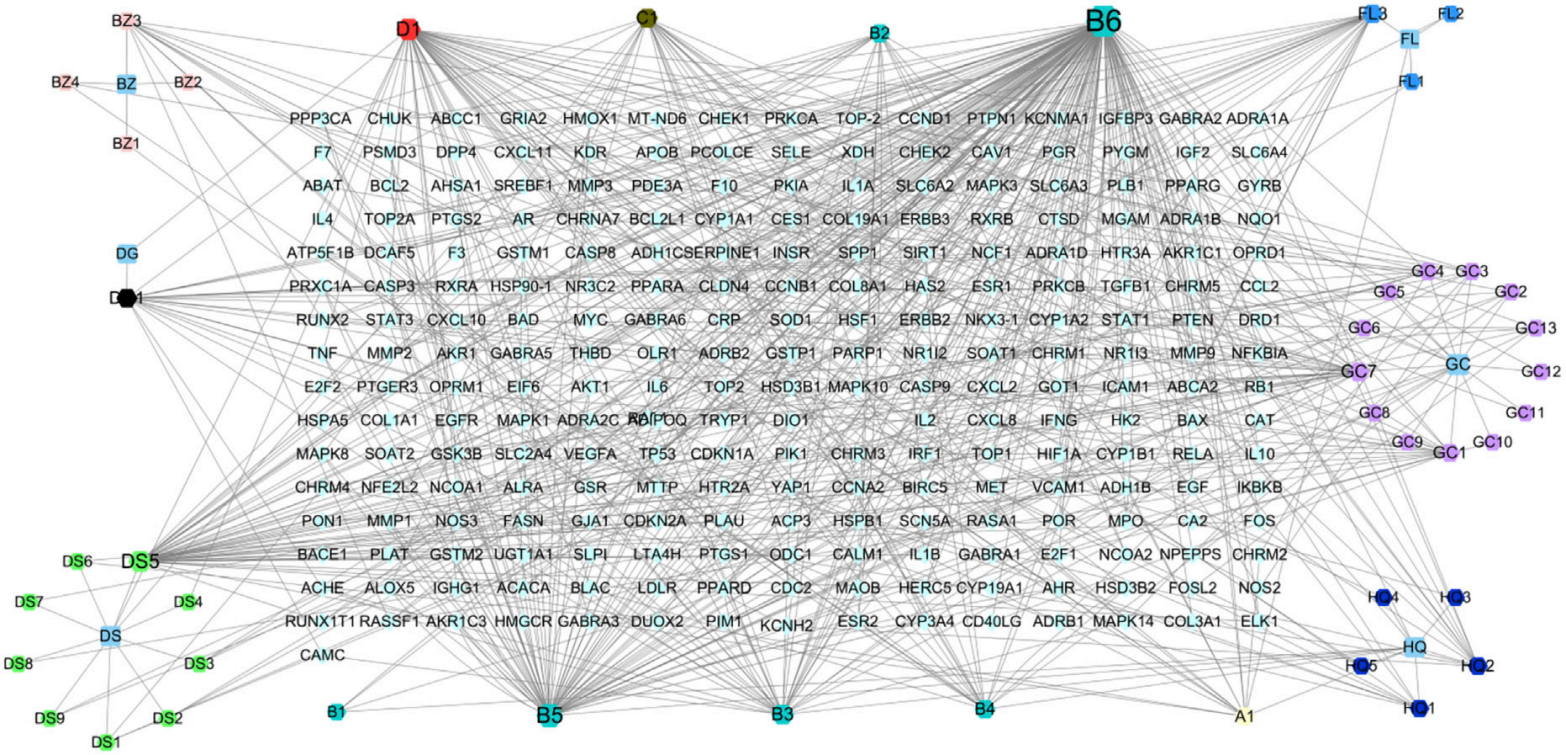
The botanical drugs-component-target diagram. The rhombus is the target, the hexagon is the effective component of botanical drug, and the square is the botanical drugs. **(A1)** refers to hederagenin, the common compound of Astragalus mongholicus Bunge Fabaceae. [Fabaceae; Astragali Radix] and Poria cocos (Schw.)Wolf. [Polyporaceae; Poria]. **(B1)** Astragalus mongholicus Bunge Fabaceae. [Fabaceae; Astragali Radix] and *Glycyrrhiza* uralensis Fisch. ex DC. [Fabaceae; Glycyrrhizae radix et rhizoma]. **(B2)** refers to Jaranol, the common compound of Astragalus mongholicus Bunge Fabaceae. [Fabaceae; Astragali Radix] and *Glycyrrhiza* uralensis Fisch. ex DC. [Fabaceae; Glycyrrhizae radix et rhizoma]. **(B3)** refers to isorhamnetin, the common compound of Astragalus mongholicus Bunge Fabaceae. [Fabaceae; Astragali Radix] and *Glycyrrhiza* uralensis Fisch. ex DC. [Fabaceae; Glycyrrhizae radix et rhizoma]. **(B4)** refers to formononetin, the common compound of Astragalus mongholicus Bunge Fabaceae. [Fabaceae; Astragali Radix] and *Glycyrrhiza* uralensis Fisch. ex DC. [Fabaceae; Glycyrrhizae radix et rhizoma]. **(B5)** refers to kaempferol, the common compound of Astragalus mongholicus Bunge Fabaceae. [Fabaceae; Astragali Radix] and *Glycyrrhiza* uralensis Fisch. ex DC. [Fabaceae; Glycyrrhizae radix et rhizoma]. **(B6)** refers to quercetin, the common compound of Astragalus mongholicus Bunge Fabaceae. [Fabaceae; Astragali Radix] and *Glycyrrhiza* uralensis Fisch. ex DC. [Fabaceae; Glycyrrhizae radix et rhizoma]. **(C1)** refers to 7-Methoxy-2-methyl isoflavone, the common compound of *Glycyrrhiza* uralensis Fisch. ex DC. [Fabaceae; Glycyrrhizae radix et rhizoma] and Codonopsis pilosula (Franch.) Nannf. [Campanulaceae; Codonopsisradix]. **(D1)** refers to Stigmasterol, the common compound of Codonopsis pilosula (Franch.) Nannf. [Campanulaceae; Codonopsisradix] and Angelica sinensis (Oliv.) Diels [Apiaceae; Angelicae Sinensis Radix].

A total of 53 effective compounds were isolated, including two *Angelica sinensis* (Oliv.) Diels (Apiaceae; Angelicae Sinensis Radix), 11 *Codonopsis pilosula* (Franch.) Nannf. (Campanulaceae; Codonopsisradix), 20 *Glycyrrhiza uralensis* Fisch. ex DC. ([Fabaceae; Glycyrrhizae radix et rhizome), four *Poria cocos* (Schw.)Wolf. (Polyporaceae; Poria), four *Atractylodes macrocephala* Koidz. (Asteraceae; Atractylodis Macrocephalae Rhizoma), and 12 *Astragalus mongholicus* Bunge Fabaceae. (Fabaceae; Astragali Radix). After removing duplicates, 44 effective compounds and 241 corresponding targets were obtained. The cytoscspe3.7.2 software was used to construct a visual interaction network diagram of “botanical drugs - components - targets” (Figure 12; Table 4).

**TABLE 4:** Degree of the key active components of botanical drugs included.

Component	Degree
*quercetin*	161
*kaempferol*	74
*Stigmasterol*	53
*luteolin*	48
*isorhamnetin*	36

#### 3.7.2 The establishments of “gastric CRF-targets” from the database and the construction of “botanical drugs-disease” venn diagram

**FIGURE 13:**
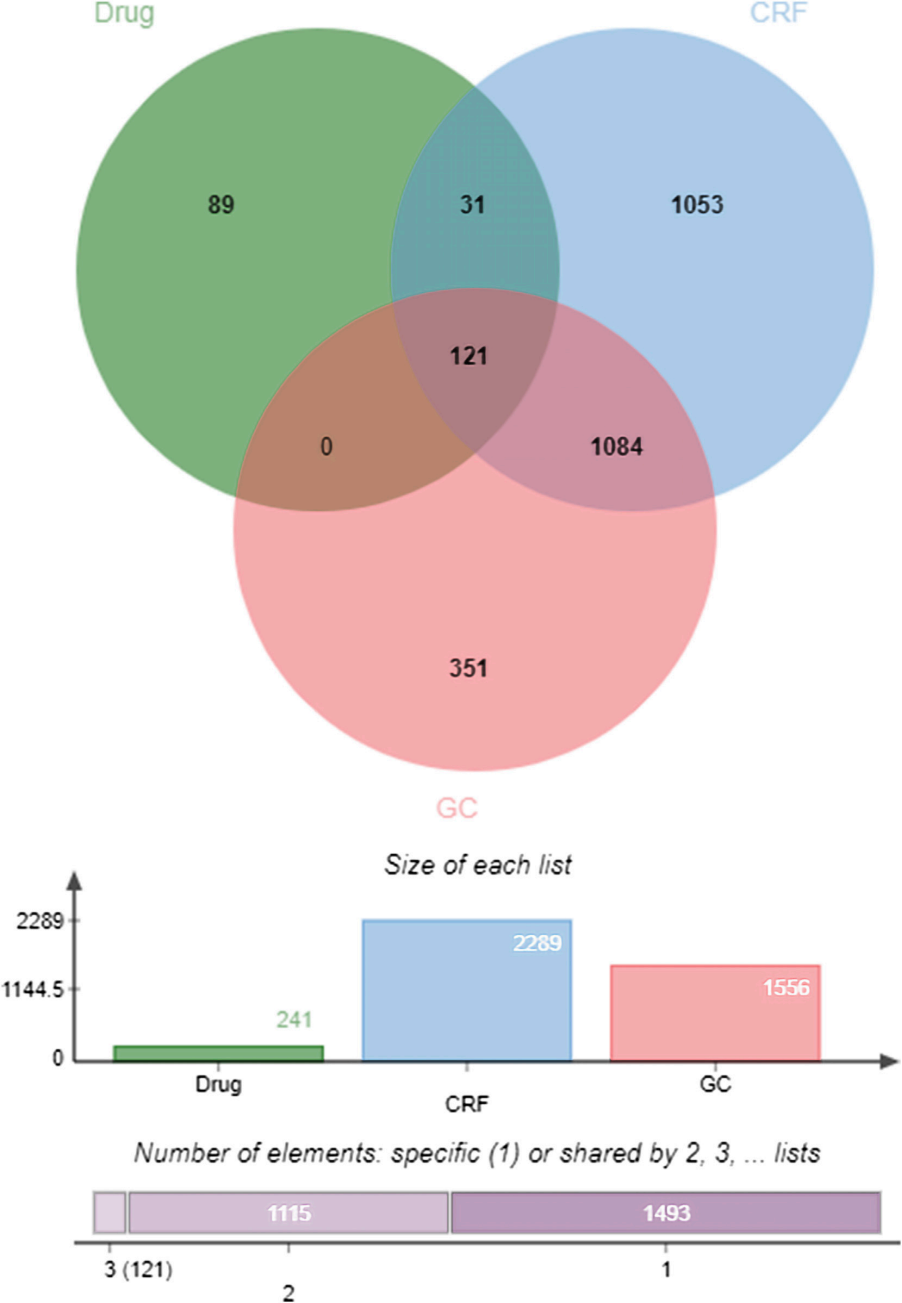
Venn diagram of “Drug—GC—CRF” targets (Drug refers to botanical drug, GC is gastric cancer, and CRF means cancer-related fatigue).

In OMIM, 574 disease targets for GC and 607 disease targets for CRF were screened, while 1,495 disease targets for GC and 1785 disease targets for CRF were found in Genecard. A total of 2,289 disease targets for CRF and 1,556 disease targets for GC were obtained after merging and de-duplication. The effective compound targets of the botanical drugs and the GC and CRF targets were isolated and intersected using the Venny2.1.0 online platform, and 121 common targets of Drug - GC—CRF were screened, as shown in Figure 13.

#### 3.7.3 The construction and analysis of PPI network

**FIGURE 14:**
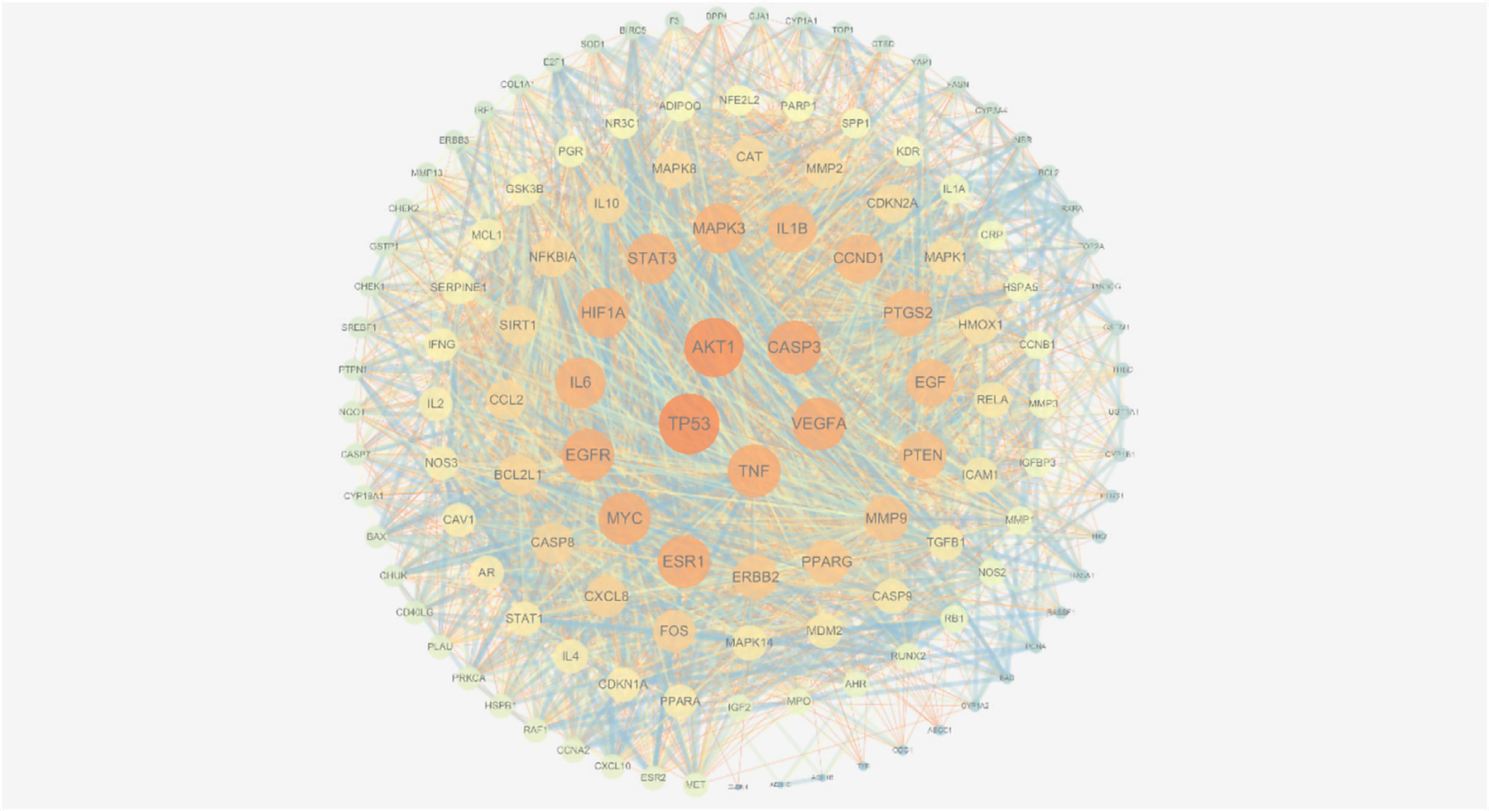
PPI network diagram of gastric CRF in botanical drugs treatment.

The TSV format file derived after importing the above 121 acting target proteins into STRING was imported into cytoscspe 3.7.2 software to obtain a key acting target network consisting of 121 nodes with 3,134 edges. Through the network topology analysis plug-in CytoNCA, five core targets and 15 major targets were obtained, as shown in Figure 14. The results showed that AKT1, TP53, TNF, VEGFA, and CASP3, the core targets, were correlated with the progression of CRF in GC and may be important in the treatment of the disease, as shown in Figure 14 and Table 5.

**TABLE 5:** Degree of the key target of botanical drugs included.

Target	Degree
*TP53*	117
*AKT1*	114
*CASP3*	102
*TNF*	100
*VEGFA*	100

#### 3.7.4 Gene Ontology (GO) enrichment analysis and KEGG enrichment analysis

**FIGURE 15:**
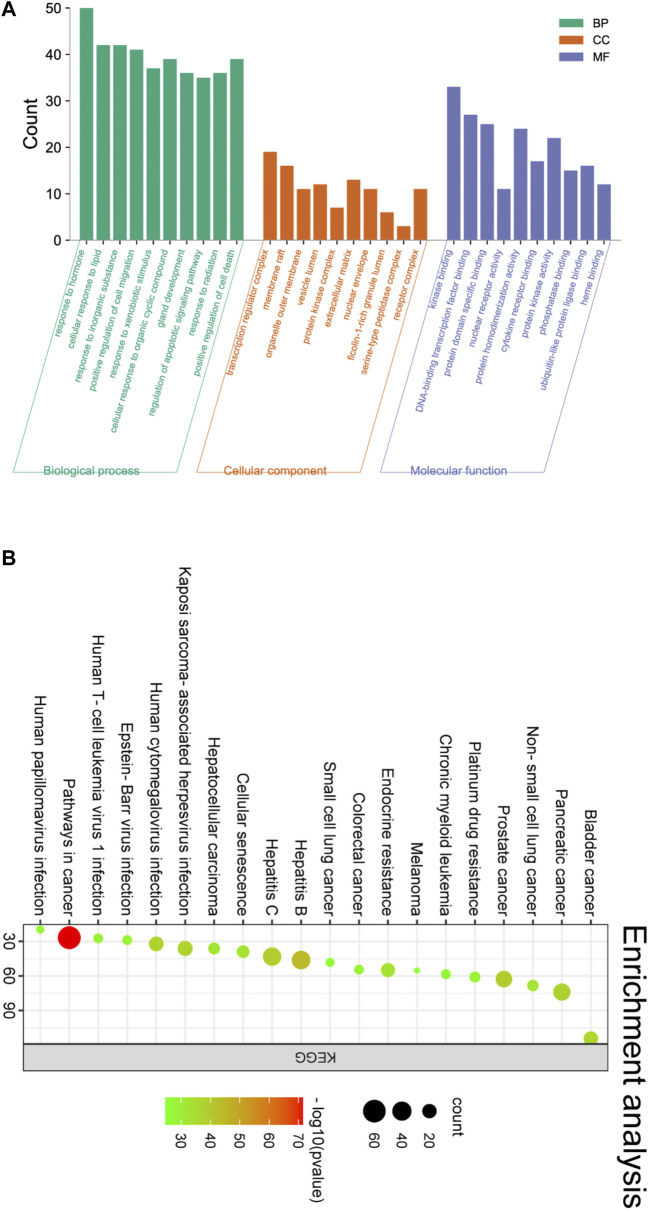
**(A)** GO functional enrichment analysis. **(B)** KEGG signaling pathway enrichment analysis.

Through GO functional enrichment analysis, 1763 BP, 65 CC, and 152 Molecular MF pathways were obtained. The top 10 BP, CC, and MF pathways were screened in ascending order of Count, and the enrichment histogram was plotted, with the main results shown in Figure 15A. A total of 190 pathways were obtained from the KEGG pathway analysis of the shared genes in Metascape. Arranged in ascending order of *p*-value, the top 20 pathways were used to map the enrichment bubble diagram, as shown in Figure 15B. The results showed that the KEGG pathways of the shared genes were mainly enriched as follows: Pathways in cancer, Hepatitis B, Prostate cancer, Hepatitis C, and Pancreatic cancer (Table 6). Most of these pathways were tumor-related, suggesting that the treatment of CRF due to GC using botanical drugs was through multiple signaling pathways.

**TABLE 6:** Information of the key pathways of botanical drug included.

Pathway	GO	Count	%	LogP
*Pathways in cancer*	hsa05200	61	47.28682171	-71.43891209
*Hepatitis B*	hsa05161	32	24.80620155	-43.95627822
*Prostate cancer*	hsa05215	26	20.15503876	-39.42753902
*Hepatitis C*	hsa05160	29	22.48062016	-38.82892583
*Pancreatic cancer*	hsa05212	24	18.60465116	-38.35228979
*Kaposi sarcoma-associated* herpesvirus *infection*	hsa05167	30	23.25581395	-37.71033118
*Human* cytomegalovirus *infection*	hsa05163	31	24.03100775	-37.3550231
*Bladder cancer*	hsa05219	20	15.50387597	-36.66107944
*Cellular senescence*	hsa04218	26	20.15503876	-33.50293177
*Endocrine resistance*	hsa01522	23	17.82945736	-33.34108111
*Non-small cell lung cancer*	hsa05223	21	16.27906977	-32.68970456
*Hepatocellular carcinoma*	hsa05225	26	20.15503876	-32.61101622
*Human T-cell leukemia virus 1 infection*	hsa05166	26	20.15503876	-29.31688318
*Colorectal cancer*	hsa05210	20	15.50387597	-28.89614904
Epstein-Barr virus *infection*	hsa05169	25	19.37984496	-28.79661502
*Platinum drug resistance*	hsa01524	19	14.72868217	-28.50078386
*Chronic myeloid leukemia*	hsa05220	19	14.72868217	-28.12462782
*Human papillomavirus infection*	hsa05165	28	21.70542636	-27.57862539
*Small cell lung cancer*	hsa05222	19	14.72868217	-26.3720016
*Melanoma*	hsa05218	17	13.17829457	-24.70804428

## 4 Discussion

CRF has a significant negative impact on the quality of life of cancer patients, especially in women (Schmidt et al., 2022). Many patients with CRF are considered to have “psychological disorders”; therefore, it is of great social and economic importance to alleviate fatigue in cancer patients. In this systematic analysis, the total CRF score, the affective, sensory, and behavioral subscales of PFS scores, the safety of botanical drugs, QLQ-C30, and KPS in the included literature, were systematically evaluated and meta-analyzed. The results showed that botanical drugs could improve all cancer-related fatigue scores and their safety profiles were satisfactory. It is noteworthy that some studies showed that the botanical drugs group had better results than the control group in gastrointestinal reactions and bone marrow suppression.

Gastric CRF is called “Xulao” or “Yuzheng” in TCM, and is believed to be caused by the growth of tumor of tumor or drug effects, resulting in the imbalance of yin and yang in the human body and the deficiency of qi and blood. Therefore, for about two thousand years, East Asian people have used drugs that can nourish Qi and blood and improve the body’s immune system in the treatment of CRF, as well as adding drugs with anti-tumor effects, such as Scutellaria barbata D.Don (Lamiaceae:Scutellariae barbatae herba) (Suh et al., 2007). This has achieved good results, which is the basis for the meta-analysis performed in this study.

Although the composition of the included groups of botanical drugs mostly varied, the frequency of use of Atractylodes macrocephala Koidz. (Asteraceae; Atractylodis Macrocephalae Rhizoma), Codonopsis pilosula (Franch.) Nannf. (Campanulaceae; Codonopsisradix), and *Glycyrrhiza* uralensis Fisch. ex DC. (Fabaceae; Glycyrrhizae radix et rhizome) was 61.5%, the frequency of use of Poria cocos (Schw.)Wolf. (Polyporaceae; Poria) was 53.8%, and these four medicines are the components of the classical botanical drugs formula Si Jun Zi Tang. Si Jun Zi Tang has the functions of tonifying Qi, promoting the vitality of the stomach, and improving the immunity of the body. Some studies have shown that Si Jun Zi Tang can induce apoptosis in GC cells (Jia et al., 2018), reduce the degree of postoperative stress and inflammatory response in gastrointestinal tumors, and enhance the immunity in patients (Liang et al., 2005). Additionally, 12 studies used Astragalus mongholicus Bunge Fabaceae. [Fabaceae; Astragali Radix] and six used Angelica sinensis (Oliv.) Diels [Apiaceae; Angelicae Sinensis Radix]. However, these form the 800-year-old botanical drug formula for improving anemia and enhancing the body’s immunity, *Angelica sinensis* decoction for supplementing blood (Danggui Buxue Decotion), which has been proven to attenuate IFN-γ-induced immune destruction of bone marrow cell hematopoiesis in modern studies (Liu et al., 2019). The most frequently used drugs is fully consistent with TCM’s understanding of spleen and gastric CRF. Therefore, a network analysis of the six drugs with the highest frequency of use among the 55 drugs used in 13 included studies was conducted to predict their potential pharmacological mechanisms.

In the network analysis of these six botanical drugs, 44 active compounds and 121 common targets of drug and gastric CRF were screened. Furthermore, 5 key active compounds, quercetin, Stigmasterol, luteolin, kaempferol and isorhamnetin as well as 5 key targets, AKT1, TP53, TNF, VEGFA and CASP3 were isolated. In addition, 5 key signaling pathways including cancer, Hepatitis B, Prostate cancer, Hepatitis C, and Pancreatic cancer pathways, were obtained through enrichment analysis. Among these, quercetin, a natural active ingredient, has been shown to induce cell morphological changes and reduce total viability through AGS apoptosis. Moreover, quercetin increased TNFRSF10D (Tumor necrosis factor receptor superfamily, member 10 days, decoy with truncated death domain) and TP53INP1 (tumor protein p53 inducible nuclear protein 1), but decreased VEGFB (vascular endothelial growth factor B) associated with the apoptotic pathway (Shang et al., 2018), which coincides with the core targets screened in the network analysis of this study. According to the KEGG enrichment analysis, botanical drugs can relieve CRF of GC through cellular senescence, which plays an important role in immunologic surveillance to ensure aging cancer cells are eliminated. Currently, cellular senescence is becoming a potential new anti-cancer strategy. It can guide effective anti-cancer treatment strategies by exploring the cell aging mode of GC (Dai et al., 2012), which also provides a direction for research on the use of botanical drugs in the treatment of CRF of GC.

This study has some limitations. First, the included literature were all in the Chinese language, and only one study mentioned the blinding of the investigators and participants (Hao and Liu, 2018); no study mentioned whether the outcome assessment was blinded and the presence of other biases. Therefore, the overall quality was low. Second, although all the included literature reported diagnostic criteria and had a pathological diagnosis as a basis, there was a lack of uniformity in the diagnostic criteria, which may lead to errors in the study results. Third, all the literature used a single-center study model, and the overall sample size was below 122; hence, there was a lack of data from multicenters and large randomized controlled trial studies. The current evidence showed that the groups of botanical drugs had better scores than the control groups. However, limited by the overall level and the number of included studies, further validation using high-quality literature is needed to confirm the above conclusions. Fourth, most of the evaluation indicators such as PFS, QLQ-C30, and KPS in the included studies were subjective, which made the study to lack the support of objective indicators. Furthermore, six most frequently used botanical drugs in the included studies were selected for network analysis in this paper; however, the network analysis only focused on the active ingredients and the targets of some single medicine without taking into account the synergistic effects of different botanical drugs. Studies have shown that Carthamus tinctorius L. (Asteraceae; Carthami flos) and Prunus persica (L.) Batsch (Rosaceae: Persicae semen) co-produce major volatile components in hot water, which are completely different from those of a single herb (Fu et al., 2012). Finally, the interventions and duration of the studies were not exactly the same, which may have influenced the results of the study.

## 5 Conclusion

In conclusion, botanical drugs therapy combined with western medicine achieved better efficacy in relieving CRF in patients with GC than western medicine alone, without increasing adverse effects. However, due to the poor quality of the included studies, more rigorously-designed multicenter and large sample-sized RCTs are needed for validation in the future. Additionally, more in-depth basic studies are required to further elucidate the pharmacological mechanisms predicted by the network analysis.

## Data Availability

The original contributions presented in the study are included in the article/Supplementary Material, further inquiries can be directed to the corresponding authors.

## Abbreviations

ADL, activities of daily living; BFI, Brit Fatigue Inventory; BP, Biological Process; CC, Cellular Component; CNKI, China National Knowledge Infrastructure; CRF, cancer-related fatigue; FDR, false discovery rate; GC, gastric cancer; GO, Gene Ontology; KEGG, Kyoto Encyclopedia of Genes and Genomes; KPS, Karnofsky Performance Status scale; MD, weighted mean difference; MF, Molecular Function; MFI, Multidimensional Fatigue Inventory; OR, odds ratio; PFS, Piper Fatigue Scale; PPI, protein-protein interaction; QLQ-C30, Quality of Life Questionnaire Core 30; RCTs, randomized controlled trials; SMD, standardized mean difference; TCM, Traditional Chinese medicine; TCMSP, Traditional Chinese Medicine Systems Pharmacology Database and Analysis Platform.
