# Supplementary material for: Effects of botanical drugs in the treatment of cancer-related fatigue in patients with gastric cancer: A meta-analysis and prediction of potential pharmacological mechanisms
Source: Front Pharmacol. 2022 Sep 7;13:979504. doi: 10.3389/fphar.2022.979504 (PMC9490126; doi:10.3389/fphar.2022.979504)
Supplement: Supplementary file 1 [file DataSheet1.PDF]

Table of contents

Supplementary File S1. PRISMA 2020 checklist..2-5

Supplementary File S2. International prospective register of systematic reviews..6-7

Supplementary File S3. Summary table of the studies include.. 8-14

Supplementary File S4. Supplementary File S4. Chemical constituents of the studies include...........................15-16

Supplementary File S5. Most commonly used ingredients in 13 study..17

Supplementary File S6. Embase Search Strategy...18-19

Supplementary File S7.The characteristics of RCTs Included in the Study...20-21

Supplementary File S8.Adverse events reported in the included studies..21-22

Supplementary File S9.Data table of Botanical Drugs components, targets and pathways were included...........23-24

Supplementary File S10.Proof of English polish...25

Supplementary File S1.PRISMA 2020 checklist

Section and Topic	Item #	Checklist item	Location where item is reported
**TITLE**			
Title	1	Identify the report as a systematic review.	P1
**ABSTRACT**			
Abstract	2	See the PRISMA 2020 for Abstracts checklist.	P2
**INTRODUCTION**			
Rationale	3	Describe the rationale for the review in the context of existing knowledge.	P3-4
Objectives	4	Provide an explicit statement of the objective(s) or question(s) the review addresses.	P3-4
**METHODS**			
Eligibility criteria	5	Specify the inclusion and exclusion criteria for the review and how studies were grouped for the syntheses.	P5
Information sources	6	Specify all databases, registers, websites, organisations, reference lists and other sources searched or consulted to identify studies. Specify the date when each source was last searched or consulted.	P4-5
Search strategy	7	Present the full search strategies for all databases, registers and websites, including any filters and limits used.	P4-5
Selection process	8	Specify the methods used to decide whether a study met the inclusion criteria of the review, including how many reviewers screened each record and each report retrieved, whether they worked independently, and if applicable, details of automation tools used in the process.	P6
Data collection process	9	Specify the methods used to collect data from reports, including how many reviewers collected data from each report, whether they worked independently, any processes for obtaining or confirming data from study investigators, and if applicable, details of automation tools used in the	P5-6

Section and Topic	Item #	Checklist item	Location where item is reported
		process.	
Data items	10a	List and define all outcomes for which data were sought. Specify whether all results that were compatible with each outcome domain in each study were sought (e.g. for all measures, time points, analyses), and if not, the methods used to decide which results to collect.	P5-6
	10b	List and define all other variables for which data were sought (e.g. participant and intervention characteristics, funding sources). Describe any assumptions made about any missing or unclear information.	P5-6
Study risk of bias assessment	11	Specify the methods used to assess risk of bias in the included studies, including details of the tool(s) used, how many reviewers assessed each study and whether they worked independently, and if applicable, details of automation tools used in the process.	P6-7
Effect measures	12	Specify for each outcome the effect measure(s) (e.g. risk ratio, mean difference) used in the synthesis or presentation of results.	P7
Synthesis methods	13a	Describe the processes used to decide which studies were eligible for each synthesis (e.g. tabulating the study intervention characteristics and comparing against the planned groups for each synthesis (item #5)).	P6-8
	13b	Describe any methods required to prepare the data for presentation or synthesis, such as handling of missing summary statistics, or data conversions.	P6-7
	13c	Describe any methods used to tabulate or visually display results of individual studies and syntheses.	P6-7
	13d	Describe any methods used to synthesize results and provide a rationale for the choice(s). If meta-analysis was performed, describe the model(s), method(s) to identify the presence and extent of statistical heterogeneity, and software package(s) used.	P6-7
	13e	Describe any methods used to explore possible causes of heterogeneity among study results (e.g. subgroup analysis, meta-regression).	P6-7
	13f	Describe any sensitivity analyses conducted to assess robustness of the synthesized results.	P6-7
Reporting bias assessment	14	Describe any methods used to assess risk of bias due to missing results in a synthesis (arising from reporting biases).	P7

Section and Topic	Item #	Checklist item	Location where item is reported
Certainty assessment	15	Describe any methods used to assess certainty (or confidence) in the body of evidence for an outcome.	P6-7
**RESULTS**			
Study selection	16a	Describe the results of the search and selection process, from the number of records identified in the search to the number of studies included in the review, ideally using a flow diagram.	P9-10
	16b	Cite studies that might appear to meet the inclusion criteria, but which were excluded, and explain why they were excluded.	P9
Study characteristics	17	Cite each included study and present its characteristics.	P9
Risk of bias in studies	18	Present assessments of risk of bias for each included study.	P13-14
Results of individual studies	19	For all outcomes, present, for each study: (a) summary statistics for each group (where appropriate) and (b) an effect estimate and its precision (e.g. confidence/credible interval), ideally using structured tables or plots.	P10-13
Results of syntheses	20a	For each synthesis, briefly summarise the characteristics and risk of bias among contributing studies.	P13-14
	20b	Present results of all statistical syntheses conducted. If meta-analysis was done, present for each the summary estimate and its precision (e.g. confidence/credible interval) and measures of statistical heterogeneity. If comparing groups, describe the direction of the effect.	P15-17
	20c	Present results of all investigations of possible causes of heterogeneity among study results.	NA
	20d	Present results of all sensitivity analyses conducted to assess the robustness of the synthesized results.	P19
Reporting biases	21	Present assessments of risk of bias due to missing results (arising from reporting biases) for each synthesis assessed.	NA
Certainty of	22	Present assessments of certainty (or confidence) in the body of evidence for each outcome assessed.	P119-20

Section and Topic	Item #	Checklist item	Location where item is reported
evidence			
**DISCUSSION**			
Discussion	23a	Provide a general interpretation of the results in the context of other evidence.	P27-30
	23b	Discuss any limitations of the evidence included in the review.	P29-30
	23c	Discuss any limitations of the review processes used.	P29-30
	23d	Discuss implications of the results for practice, policy, and future research.	P27-30
**OTHER INFORMATION**			
Registration and protocol	24a	Provide registration information for the review, including register name and registration number, or state that the review was not registered.	P4
	24b	Indicate where the review protocol can be accessed, or state that a protocol was not prepared.	P4
	24c	Describe and explain any amendments to information provided at registration or in the protocol.	P4
Support	25	Describe sources of financial or non-financial support for the review, and the role of the funders or sponsors in the review.	P31
Competing interests	26	Declare any competing interests of review authors.	P31
Availability of data, code and other materials	27	Report which of the following are publicly available and where they can be found: template data collection forms; data extracted from included studies; data used for all analyses; analytic code; any other materials used in the review.	P31

From: Page MJ, McKenzie JE, Bossuyt PM, Boutron I, Hoffmann TC, Mulrow CD, et al. The PRISMA 2020 statement: an updated guideline for reporting systematic reviews. BMJ 2021;372:n71. doi: 10.1136/bmj.n71

For more information, visit: http://www.prisma-statement.org/

Supplementary File S2.International prospective register of systematic reviews

To enable PROSPERO to focus on COVID-19 submissions, this registration record has undergone basic automated checks for eligibility and is published exactly as submitted. PROSPERO has never provided peer review, and usual checking by the PROSPERO team does not endorse content. Therefore, automatically published records should be treated as any other PROSPERO registration. Further detail is provided here.

Citation

WANG Ziming, Shasha Mei. Qing Xiang, Zhenzhong Xia, Zihong Wu, Aohan Hao. Efficacy and safety of Chinese Medicine on cancer-related fatigue for stomach neoplasms patients: A systematic review and meta-analysis. PROSPERO 2022 CRD42022324654 Available from:
https://www.crd.york.ac.uk/prospero/display_record.php?ID=CRD42022324654

Review question

To explore the clinical effectiveness of traditional Chinese medical therapy for treating cancer-related fatigue for stomach neoplasms by searching randomized controlled studies.

To explore the safety of traditional Chinese medical therapy for treating cancer-related fatigue for stomach neoplasms by searching randomized controlled studies.

Searches

Seven different databases (China National Knowledge Infrastructure. Wanfang Database. VIP. China Biological Medicine Database, PubMed, Cochrane Library and Embase) were independently searched from inception to April 2022. The search terms used following: (Stomach Neoplasms OR Gastric Neoplasms OR Stomach Cancers) AND (cancer-related fatigue OR fatigue) AND Chinese medical. Two evaluators independently screened the literature, extracted and cross-checked the data. The third party was consulted for assistance in judgment, when evaluators had disagreements.

Types of study to be included

Randomized controlled trials (RCTs) of Chinese medical therapy treatment for treating cancer-related fatigue for stomach neoplasms will be included in this review. as either full articles or abstracts.

Condition or domain being studied

Cancer-related fatigue (CRF) is defined by the National Comprehensive Cancer Network (NCCN) as a "persistent, subjective sense of tiredness related to cancer and cancer treatment that interferes with usual functioning". CRF considerably affects the functional status and health-related quality of life (HRQoL) of cancer patients with reduction of ability at physical. mental, emotional or social levels. The effectiveness of treatment for CRF In NCCN is hardly satisfactory.

Traditional Chinese medical therapy has been widely used in the treatment of people with cancer in China and other eastern countries. It includes traditional Chinese medicine (TCM), acupuncture, moxibustion, massage and other traditional Chinese medical therapies. Relevant studies found that traditional Chinese medical therapy has potential beneficial effects on assisting in treating cancer, retarding cancer progression, boosting immune system, ameliorating chemotherapy or radiotherapy-induced complications and side-effects, such as pain and fatigue.

Participants/population

People with a clinical diagnosis of cancer-related fatigue for stomach will be included.

There will be no age. gender, cancer stage or type of cancer treatment restrictions.

Intervention(s), exposure(s)

We will include those trials on traditional Chinese medical therapy used alone or as combined therapies of traditional Chinese medical treatments with conventional therapies versus the same conventional therapies.Chinese herbal medicine is defined as single herb, Chinese patent medicine, individually

NIHR | National Institute for Health Research
PROSPERO
International prospective register of systematic reviews

prescribed herbal formulae and herbal products extracted from natural herbs. There are no limitations on the number of herbs used, the dosages, the administration, or the duration of the treatment.

Comparator(s)/control

We will include trials comparing traditional Chinese medical therapy with sham (placebo), no treatment and other active non-TCM therapy (e.g. education, physical therapy or medication). The trials in which one form of TCM therapy has been compared with another form of TCM therapy will be excluded.

Main outcome(s)

Cancer-related fatigue using validated scales for assessment.

Additional outcome(s)

These will include QoL measured by validated instruments, improvement of depression or anxiety, and adverse events related to traditional Chinese medical therapy.

Data extraction (selection and coding)

(1) Basic information of studies: study title, author name and publication year; (2) Baseline characteristics of studies: sample size, age, gender and diseases; (3) Intervention measures and follow-up time; (4) Outcome data.

Risk of bias (quality) assessment

The Cochrane Risk Bias Assessment Tool was used as the standard for rigorous evaluation as following aspects: (1) method of random allocation and allocation concealment; (2) blinding method of participants and personnel; (3) blinding method of outcome assessment; (4) selectivity of result reporting; (5) other biases.

Strategy for data synthesis

Data analysis:

The results of comparable groups of studies will be performed with RevMan 5.3 software to compare the efficacy outcomes of traditional Chinese medical therapy versus the control group. If the homogeneity of the trials based on study design, participants, interventions, controls, and outcome measures are acceptable, meta-analysis will be performed. We will adopt a fixed-effects model for the meta-analysis for non-significant heterogeneity and a random-effects model for significant heterogeneity ($p<0.1$).

Dealing with missing data:

Where there are missing data, we will investigate the reasons for its absence. Whenever possible, we will contact the original study authors to request any missing data by e-mail. If it is possible, we will also conduct intention-to-treat(ITT) analysis.

Sensitivity analysis and publication bias:

We plan to conduct a sensitivity analysis to explore the influence of trial quality on effect estimates if a sufficient number of randomized trials are found. Publication bias will be demonstrated by applying a funnel plot analysis if more than ten trials are identified.

Analysis of subgroups or subsets

If there is an adequate number of studies, we will conduct subgroup analysis to interpret the heterogeneity between studies, as follows:

1. Type of Chinese medical (e.g. single herb, Chinese patent medicine, individually prescribed herbal formulae and herbal products extracted from natural herbs).

2. Type of control (e.g. sham/placebo, conventional/usual care or no treatment).

3. Whether or not participants are receiving chemotherapy, radiation therapy or other relevant Western therapies.

Contact details for further information
WANG Ziming
254806326@qq.com

Organisational affiliation of the review
Hubei University of Chinese Medicine

Review team members and their organisational affiliations
Mr WANG Ziming. Hubei University of Chinese Medicine
Dr Shasha Mei. Renmin Hospital of Wuhan University
Dr Qing Xiang. Renmin Hospital of Wuhan University
Mr Zhenzhong Xia. Hubei University of Chinese Medicine
Dr Zihong Wu. Chengdu University of Traditional Chinese Medicine
Miss Aohan Hao. Hubei University of Chinese Medicine

Type and method of review
Meta-analysis, Systematic review

Anticipated or actual start date
10 April 2022

Anticipated completion date
20 May 2022

Funding sources/sponsors
TCM Scientific research project of Hubei Provincial Health Commission

Conflicts of interest

Language
English

Country
China

Stage of review
Review Ongoing

Subject index terms status
Subject indexing assigned by CRD

Subject index terms
MeSH headings have not been applied to this record

Date of registration in PROSPERO
10 May 2022

Date of first submission
10 April 2022

Stage of review at time of this submission

NIHR | National Institute for Health Research
PROSPERO
International prospective register of systematic reviews

Stage	Started	Completed
Preliminary searches	Yes	Yes
Piloting of the study selection process	Yes	No
Formal screening of search results against eligibility criteria	No	No
Data extraction	No	No
Risk of bias (quality) assessment	No	No
Data analysis	No	No

The record owner confirms that the information they have supplied for this submission is accurate and complete and they understand that deliberate provision of inaccurate information or omission of data may be construed as scientific misconduct.
The record owner confirms that they will update the status of the review when it is completed and will add publication details in due course.

Versions
10 May 2022
10 May 2022

Supplementary File S3. Summary table of the studies include.

Study	Species, source, concentration	Quality control reported? (Y/N)	Chemical analysis reported? (Y/N)
Wang 2018	*Codonopsis pilosula (Franch.) Nannf. [Campanulaceae;Codonopsisradix]*<br>*Astragalus mongholicus Bunge Fabaceae.[Fabaceae;Astragali Radix]*	Y–<br>Chinese medicine injection	Y – HPLC or other analytical system
Ma et al *2020*	*Astragalus mongholicus Bunge Fabaceae.[Fabaceae;Astragali Radix] 30g*<br>*Codonopsis pilosula (Franch.) Nannf. [Campanulaceae;Codonopsisradix] 20g*<br>*Poria cocos（Schw.）Wolf. [Polyporaceae;Poria]20g*<br>*Atractylodes macrocephala Koidz. [Asteraceae;Atractylodis Macrocephalae Rhizoma]20g*<br>*Angelica sinensis (Oliv.) Diels [Apiaceae；Angelicae Sinensis Radix]10g*<br>*Paeonia lactiflora Pall. [Paeoniaceae：paeoniae radix alba]10g*<br>*Artemisia annua L. [Asteraceae：artemisiae annuae herba]10g*<br>*Citrus × aurantium L. [Rutaceae：citri reticulatae pericarpium]10g*<br>*Dolomiaea costus (Falc.) Kasana & A.K.Pandey [Asteraceae：aucklandiae radix]10g*<br>*Glycyrrhiza uralensis Fisch. ex DC. [Fabaceae;Glycyrrhizae radix et*	Y–<br>Hospital preparation，Decoction	N

	rhizoma]10g		
Hao etal 2018	*Astragalus mongholicus Bunge Fabaceae.[Fabaceae;Astragali Radix]30g*<br>*Codonopsis pilosula (Franch.) Nannf. [Campanulaceae;Codonopsisradix]15g*<br>*Atractylodes macrocephala Koidz. [Asteraceae;Atractylodis Macrocephalae Rhizoma]15g*<br>*Poria cocos（Schw.）Wolf. [Polyporaceae;Poria]15g*<br>*Angelica sinensis (Oliv.) Diels [Apiaceae；Angelicae Sinensis Radix]15g*<br>*Rehmannia glutinosa (Gaertn.) DC. [Orobanchaceae:Rehmanniae radix]15g*<br>*Polygonatum sibiricum Redouté [Asparagaceae：Polygonati rhizoma]15g*<br>*Ophiopogon japonicus (Thunb.) Ker Gawl. [Asparagaceae：Ophiopogonis radix]15g*<br>*Dendrobium nobile Lindl. [Orchidaceae：Dendrobii caulis]10g*<br>*Citrus × aurantium L. [Rutaceae：citri reticulatae pericarpium]10g*<br>*Pinellia ternata (Thunb.) Makino [Araceae：Pinelliae rhizoma]10g*<br>*Zingiber officinale Roscoe [Zingiberaceae：Zingiberis rhizoma]10g*<br>*Hordeum vulgare L. [Poaceae：Hordei fructus germinatus]15g*<br>*Setaria italica (L.) P.Beauv. [Poaceae：Setariae fructus germinatus]15g*<br>*Ziziphus jujuba Mill. [Rhamnaceae：Ziziphi spinosae semen]15g*<br>*Platycladus orientalis (L.) Franco [Cupressaceae：Platycladi semen]6g*<br>*Schisandra chinensis (Turcz.) Baill. [Schisandraceae:Schisandrae chinensis fructus]6g*<br>*Glycyrrhiza uralensis Fisch. ex DC. [Fabaceae;Glycyrrhizae radix et rhizoma]6g*	Y−<br>Hospital preparation，Decoction	N

Zhu et al 2019	*Pseudostellaria heterophylla (Miq.) Pax [Caryophyllaceae ： Pseudostellariae radix]30g*<br>*Astragalus mongholicus Bunge Fabaceae.[Fabaceae;Astragali Radix] 30g*<br>*Atractylodes macrocephala Koidz. [Asteraceae;Atractylodis Macrocephalae Rhizoma]12g*<br>*Poria cocos（Schw.）Wolf. [Polyporaceae;Poria]15g*<br>*Rehmannia glutinosa (Gaertn.) DC. [Orobanchaceae ： Rehmanniae radix]30g*<br>*Angelica sinensis (Oliv.) Diels [Apiaceae；Angelicae Sinensis Radix]12g*<br>*Cuscuta chinensis Lam. [Convolvulaceae：Cuscutae semen]12g*<br>*Lycium barbarum L. [Solanaceae：Lycii fructus]15g*<br>*Gynostemma pentaphyllum (Thunb.) Makino [Cucurbitaceae ： Gynostemma]15g*<br>*Atractylodes lancea (Thunb.) DC. [Asteraceae：Atractylodis rhizoma]9g*<br>*Citrus medica L. [Rutaceae：Citri sarcodactylis fructus]12g*<br>*Glycyrrhiza uralensis Fisch. ex DC. [Fabaceae;Glycyrrhizae radix et rhizoma]*	Y–<br>Hospital preparation，Decoction	N
Li 2020	*Mylabris phalerata Pallas[Meloidae：Mylabris phalerata Pallas]*<br>*Panax ginseng C.A.Mey. [Araliaceae：Ginseng radix et rhizoma]*<br>*Astragalus mongholicus Bunge Fabaceae.[Fabaceae;Astragali Radix]*<br>*Eleutherococcus senticosus (Rupr. & Maxim.) Maxim. [Araliaceae ： Acanthopanacis senticosi radix et rhizoma seu caulis]*	Y–<br>Chinese medicine injection	Y – HPLC or other analytical system
Si et al 2019	*Mylabris phalerata Pallas[Meloidae：Mylabris phalerata Pallas]*<br>*Panax ginseng C.A.Mey. [Araliaceae：Ginseng radix et rhizoma]*<br>*Astragalus mongholicus Bunge Fabaceae.[Fabaceae;Astragali Radix]*<br>*Eleutherococcus senticosus (Rupr. & Maxim.) Maxim. [Araliaceae ：*	Y–<br>Chinese medicine injection	Y – HPLC or other analytical system

	Acanthopanacis senticosi radix et rhizoma seu caulis]		
Chen et al 2019	*Codonopsis pilosula (Franch.) Nannf. [Campanulaceae;Codonopsisradix]15g*<br>*Poria cocos（Schw.）Wolf. [Polyporaceae;Poria]15g*<br>*Rehmannia glutinosa (Gaertn.) DC. [Orobanchaceae；Rehmanniae radix]15g*<br>*Paeonia lactiflora Pall. [Paeoniaceae；paeoniae radix alba]15g*<br>*Angelica sinensis (Oliv.) Diels [Apiaceae；Angelicae Sinensis Radix]15g*<br>*Atractylodes macrocephala Koidz. [Asteraceae;Atractylodis Macrocephalae Rhizoma]12g*<br>*Conioselinum anthriscoides 'Chuanxiong' [Apiaceae；Chuanxiong rhizoma]10g*<br>*Glycyrrhiza uralensis Fisch. ex DC. [Fabaceae;Glycyrrhizae radix et rhizoma]10g*<br>*Astragalus mongholicus Bunge Fabaceae.[Fabaceae;Astragali Radix]10g*	Y–<br>Hospital preparation，Decoction	N
Wang et al 2016	*Atractylodes macrocephala Koidz. [Asteraceae;Atractylodis Macrocephalae Rhizoma]10g*<br>*Angelica sinensis (Oliv.) Diels [Apiaceae；Angelicae Sinensis Radix]10g*<br>*Poria cocos（Schw.）Wolf. [Polyporaceae;Poria]15g*<br>*Astragalus mongholicus Bunge Fabaceae.[Fabaceae;Astragali Radix]10g*<br>*Polygala senega L. [Polygalaceae；Polygalae radix]15g*<br>*Dimocarpus longan Lour. [Sapindaceae；Longan arillus]10g*<br>*Ziziphus jujuba Mill. [Rhamnaceae；Ziziphi spinosae semen]15g*<br>*Panax ginseng C.A.Mey. [Araliaceae；Ginseng radix et rhizoma]10g*<br>*Dolomiaea costus (Falc.) Kasana & A.K.Pandey [Asteraceae：aucklandiae*	Y–<br>Hospital preparation，Decoction	N

	radix]15g<br>*Glycyrrhiza uralensis Fisch. ex DC. [Fabaceae;Glycyrrhizae radix et rhizoma]5g*<br>*Zingiber officinale Roscoe [Zingiberaceae；Zingiberis rhizoma recens]10g*<br>*Ziziphus jujuba Mill. [Rhamnaceae；Jujubae fructus]10g*		
Gao 2019	*Codonopsis pilosula (Franch.) Nannf. [Campanulaceae;Codonopsisradix]*<br>*Astragalus mongholicus Bunge Fabaceae.[Fabaceae;Astragali Radix]*	Y–<br>Chinese medicine injection	Y – HPLC or other analytical system
Fan et al 2015	*Astragalus mongholicus Bunge Fabaceae.[Fabaceae;Astragali Radix]20g*<br>*Codonopsis pilosula (Franch.) Nannf. [Campanulaceae;Codonopsisradix]15g*<br>*Atractylodes macrocephala Koidz. [Asteraceae;Atractylodis Macrocephalae Rhizoma]10g*<br>*Poria cocos（Schw.）Wolf. [Polyporaceae;Poria]10g*<br>*Cyperus rotundus L. [Cyperaceae；Cyperi rhizoma]10g*<br>*Curcuma longa L. [Zingiberaceae；Curcumae radix]10g*<br>*Biancaea sappan (L.) Tod. [Fabaceae；Sappan lignum]10g*<br>*Curcuma longa L. [Zingiberaceae；Curcumae rhizoma]10g*<br>*Paris polyphylla var. yunnanensis (Franch.) Hand.-Mazz. [Melanthiaceae；Paridis rhizoma]9g*<br>*Scutellaria barbata D.Don [Lamiaceae：Scutellariae Barbatae Herba]10g*<br>*Ligustrum lucidum W.T.Aiton [Oleaceae；Ligustri lucidi fructus]10g*<br>*Cuscuta chinensis Lam. [Convolvulaceae；Cuscutae semen]10g*<br>*Hordeum vulgare L. [Poaceae；Hordei fructus germinatus]15g*<br>*Setaria italica (L.) P.Beauv. [Poaceae；Setariae fructus germinatus]15g*<br>*Gallus gallus domesticus Brisson[Phasianidae；Galli gigerii endothelium*	Y–<br>Hospital preparation，Decoction	N

	corneum]9g<br>*Glycyrrhiza uralensis Fisch. ex DC. [Fabaceae;Glycyrrhizae radix et rhizoma]5g*		
Ai et al 2021	*Mylabris phalerata Pallas[Meloidae：Mylabris phalerata Pallas]*<br>*Panax ginseng C.A.Mey. [Araliaceae：Ginseng radix et rhizoma]*<br>*Astragalus mongholicus Bunge Fabaceae.[Fabaceae;Astragali Radix]*<br>*Eleutherococcus senticosus (Rupr. & Maxim.) Maxim. [Araliaceae：Acanthopanacis senticosi radix et rhizoma seu caulis]*	Y–<br>Chinese medicine injection	Y – HPLC or other analytical system
Yong et al 2021	*Codonopsis pilosula (Franch.) Nannf. [Campanulaceae;Codonopsisradix]30g*<br>*Poria cocos（Schw.）Wolf. [Polyporaceae;Poria]15g*<br>*Atractylodes macrocephala Koidz. [Asteraceae;Atractylodis Macrocephalae Rhizoma]15g*<br>*Dioscorea oppositifolia L. [Dioscoreaceae；Dioscoreae rhizoma]15g*<br>*Salvia miltiorrhiza Bunge [Lamiaceae； Salviae miltiorrhizae radix et rhizoma]15g*<br>*Lycium barbarum L. [Solanaceae；Lycii fructus]15g*<br>*Cuscuta australis R.Br. [Convolvulaceae；Cuscutae semen]10g*<br>*Pinellia ternata (Thunb.) Makino [Araceae；Pinelliae rhizoma]6g*<br>*Glycyrrhiza uralensis Fisch. ex DC. [Fabaceae;Glycyrrhizae radix et rhizoma]9g*	Y–<br>Hospital preparation，Decoction	N
Gu et al 2021	*Astragalus mongholicus Bunge Fabaceae.[Fabaceae;Astragali Radix]30g*<br>*Cullen corylifolium (L.) Medik. [Fabaceae；Psoraleae fructus]15g*<br>*Glycyrrhiza uralensis Fisch. ex DC. [Fabaceae； Glycyrrhiza uralensis Fisch. ex DC. [Fabaceae;Glycyrrhizae radix et rhizoma]]15g*<br>*Epimedium sagittatum (Siebold & Zucc.) Maxim. [Berberidaceae；*	Y–<br>Hospital preparation，Decoction	N

	Epimedii folium]15g<br>*Cuscuta australis R.Br. [Convolvulaceae；Cuscutae semen]15g*<br>*Lycium barbarum L. [Solanaceae；Lycii fructus]15g*<br>*Bupleurum scorzonerifolium Willd. [Apiaceae；Bupleuri radix]10g*<br>*Atractylodes macrocephala Koidz. [Asteraceae;Atractylodis Macrocephalae Rhizoma]10g*<br>*Citrus × aurantium L. [Rutaceae；citri reticulatae pericarpium]10g*<br>*Codonopsis pilosula (Franch.) Nannf. [Campanulaceae;Codonopsisradix]10g*<br>*Pinellia ternata (Thunb.) Makino [Araceae；Pinelliae rhizoma]10g*<br>*Actaea heracleifolia (Kom.) J.Compton [Ranunculaceae；Cimicifugae rhizoma]6g*<br>*Scutellaria baicalensis Georgi [Lamiaceae；Scutellariae radix]6g*<br>*Angelica sinensis (Oliv.) Diels [Apiaceae；Angelicae Sinensis Radix]6g*		

Supplementary File S4. Chemical constituents of the studies include.

Chemical constituents of Aidi Injection(Ai et al 2021,Li 2020,Si et al 2019)

Twenty-two compounds were isolated and identified as 3-O-(3′, 4′-diacetyl)-β-Dxylopyranosyl-6-O-β-D-glucopyranosyl-cycloastragenol (1), astragaloside IV (2), astragaloside II (3), astragaloside I (4), isoastragaloside I (5), acetylastragaloside I (6), ginsenosid-Re (7), ginsenoside-Rf (8), ginsenoside-Rg1 (9), ginsenoside-Rb3 (10), notoginsenoside-R4 (11), ginsenoside-Rb1 (12), ginsenoside-Rc (13), ginsenoside-Rb2 (14), ginsenoside-Rd (15), lucyoside H (16), 3-O-β-D-glucopyranosyl (1→4)-β-D-glucopyranosyl (1→3)-α-L-rhamnopyranosyl (1→2)-α-L-arabinopyranosyl oleanolic acid 28-O--L-rhamnopyranosyl (1→4)-β-D-glucopyranosyl (1→6)-β-D-glucopyranoside (17), 3-O-β-D-glucopyranosyl (1→3)-α-L-rhamnopyranosyl [β-D-glucopyranosyl-(1→4)]-(1→2)-α-L-arabinopyranosyl oleanolic acid 28-O-α-L-arabinopyranosyl (1→4)-β-D-rhamnopyranosyl (1→6)-β-D-glucopyranoside (18), syringin (19), elentheroside E (20), 4-(1, 2, 3-trihydroxypropyl)-2, 6-dimethoxyphenyl1-O-β-D-glucopyranoside (21), and coniferin (22).

ZHANG,M.,LIU,Y.L.,LI,X.R.,XU,Q.M.&YANG,S.L.(2012),"Studies on chemical constituents from Aidi Injection",*Chinese Traditional and Herbal Drugs*,Vol. 43 No. 08,pp.1462-1470.

Chemical constituents of Shenqi Fuzheng Injection(Gao 2019,Wang 2018,Chen et al 2019)

Fourteen compounds were isolated，which were identified as calycosin (1) ，calycosin 7-O-β-D-glucopyranoside (2) ，formononetin 7-O-β-D-glucopyranoside (3) ，(6aR ， 11aR) -9 ， 10-dimethoxypeterocarpan-3-O-β-D-glucopyranoside (4) ， syringin(5) ， 5-hydroxymethyl-2-furfural (6) ，(3R) -(-) -2'-hydroxyl-3'，4'-dimethoxyisoflavan-7-O-β-D-glucopyranoside (7) ，(6aR，11aR) -vesticarpan (8) ，glucosyringic acid (9) ，(+) -syringaresinol 4-O-β-D-glucopyranoside (10) ，(Z) -cinnamic acid 8-O-β-D-glucopyranoside (11) ， guanosine (12) ，adenosine (13) ，and vanillic acid (14).

ZHANG,S.Y.,FAN,C.L.,WANG,L.,LIU,X.H.,SUN,X.W.&YE,W.C.(2011),"Chemical constituents of Shenqi Fuzheng Injection",*Chinese Traditional Patent Medicine*,Vol. 33 No. 10,PP.1743-1747.

Supplementary File S5. Most commonly used ingredients in 13 study.

Chinese name	Pharmaceutical name	Species	Family	N/13(%)
Huangqi	Astragali Radix	*Astragalus mongholicus Bunge*	Fabaceae	12(92.3%)
Baizhu	Atractylodis Macrocephalae Rhizoma	*Atractylodes lancea (Thunb.) DC.*	Asteraceae	8(61.5%)
Dangshen	Codonopsisradix	*Codonopsis pilosula (Franch.) Nannf.*	*Campanulac-eae*	8(61.5%)
Gancao	Glycyrrhizae radix et rhizoma	*Glycyrrhiza uralensis Fisch. ex DC.*	*Fabaceae*	8(61.5%)
Fuling	Poria	*Brassica napus L.*	Brassicaceae	7(53.8%)
Danggui	Angelicae Sinensis Radix	*Angelica sinensis (Oliv.) Diels*	Apiaceae	6(46.1%)

Supplementary File S6.Embase Search Strategy

#1'**stomach tumor**'/exp
#2'**neoplasm, stomach**':ab,ti
#3'**stomach neoplasms**':ab,ti
#4'**stomach neoplasm**':ab,ti
#5'**neoplasms, stomach**':ab,ti
#6'**gastric neoplasms**':ab,ti
#7'**gastric neoplasm**':ab,ti
#8'**neoplasm, gastric**':ab,ti
#9'**neoplasms, gastric**':ab,ti
#10'**cancer of stomach**':ab,ti
#11'**stomach cancers**':ab,ti
#12'**gastric cancer**':ab,ti
#13'**cancer, gastric**':ab,ti
#14'**cancers, gastric**':ab,ti
#15'**gastric cancers**':ab,ti
#16'**stomach cancer**':ab,ti
#17'**cancer, stomach**':ab,ti
#18'**cancers, stomach**':ab,ti
#19'**cancer of the stomach**':ab,ti
#20'**gastric cancer, familial diffuse**':ab,ti
#21#1 OR #2 OR #3 OR #4 OR #5 OR #6 OR #7 OR #8 OR #9 OR #10 OR #11 OR #12 OR #13 OR #14 OR #15 OR#16OR#17 OR #18 OR #19 OR #20
#22'**fatigue**':ab,ti
#23'**cancer related fatigue**':ab,ti

#24'crf':ab,ti
#25#22 OR #23 OR #24
#26'chinese medicine'/exp
#27'drugs':ab,ti
#28'chinese herbal':ab,ti
#29'chinese drugs':ab,ti
#30'plant':ab,ti
#31'chinese herbal drugs':ab,ti
#32'chinese herbal drugs':ab,ti
#33'plant extracts, chinese':ab,ti
#34'chinese plant extracts':ab,ti
#35'extracts, chinese plan':ab,ti
#36'medicine, chinese traditional':ab,ti
#37'herbal medicine':ab,ti
#38'trditional chinese medicine':ab,ti
#39'traditional oriental medicine':ab,ti
#40'herb':ab,ti
#41'decoction':ab,ti
#42'botanic':ab,ti
#43'injection':ab,ti
#44#22 AND #23 AND #24 AND #25 AND #26 AND #27 AND #28 AND #29 AND #30 AND #31 AND #32 AND #33 AND #34 AND #35 AND #36 AND #37AND #38 AND #39 AND #40 AND #41 AND #42 AND #43
#45'random' OR **'placebo'** OR **'double-blind'**:ab,ti
#46#21 AND #25 AND #44 AND #45

Supplementary File S7.The characteristics of RCTs Included in the Study

Study ID	Region	Sample size (T/C)	Age (y)		Gender (M/F)		Intervention		Duration	Outcome
			T	C	T	C	T	C	(weeks)	
Wang 2018	China	20/20	65.9±10.198	64.4±12.713	12/8	13/7	SOX +Shenqi Fuzheng Injection	SOX	3	a,b,c,d,f,
Ma etal 2020	China	30/30	63.±5.6	62.3±4.3	16/14	17/13	SOX +Xingjian Decoction	SOX	9	a,c,d,
Hao etal 2018	China	38/38	64.02±9.15	61.61±10.20	24/14	20/17	Yiqi Yangxue decoction +Basic treatment to symptoms	Basic treatment to symptoms	NA	a
Zhu etal 2019	China	61/61	62.58±9.33	61.27±11.35	30/31	32/29	FOLFOX4 + Jianpiyishen Formula	FOLFOX4	8	a,b,c,d,e,f
Li 2020	China	32/30	67.20±6.92	66.74±7.35	22/10	19/11	SOX +Aidi Injection	SOX	12	a,g
Si etal 2019	China	60/56	58.5± 12.5	58.2± 11.6	37/23	35/21	Oxaliplatin and capecitabine chemotherapy+Aidi injection	Oxaliplatin and capecitabine chemotherapy	2	a,b,c,e
Chen etal 2019	China	30/30	52.13±4.17	52.13±4.17	15/15	15/15	FOLFOX4+Bazhen decoction+Shenqi Fuzheng Injection	FOLFOX4	4	a,b,c,d,e

Wang etal 2016	China	42/42	65-77	66-80	33/9	32/10	Tegio capsule+guipi decoction	Tegio capsule	6	a
Gao 2019	China	30/30	NA	NA	26/4	23/7	SOX + Shenqi Fuzheng Injection	SOX	6	a,e
Fan etal 2015	China	30/30	52.73 ± 1. 92	51. 07 ± 1. 85	15/15	14/16	Weifu Formula +FOLFOX6	FOLFOX6	6	a
Ai etal 2021	China	35/36	53.03 ±2.24	53.85 ±2.09	18/17	19/17	SOX+Aidi Injection	SOX	12	a
Gu et al 2021	China	30/30	62.53±5.84	63.07±5.90	21/9	18/12	Buzhong yishen decoction+Basic treatment to symptoms	Basic treatment to symptoms	4	a,e
Yong et al 2021	China	60/60	55.43±6.94	54.26±6.74	28/20	36/21	ECF+Jianpi Kangai decoction	ECF	9	a,f

Characteristics of RCTs included in the study.

T, treatment group; C, control group; NA, not available; M/F, male/female; Outcomes: a, CRF total score; b, affective subscales of PFS scores*; c,* sensory subscales of PFS scores*; d,* behavioral subscales of PFS scores*; e, QLQ-C30 score; f, KPS score; SOX,SOX chemotherapy regimens; FOLFOX4 ,FOLFOX4 chemotherapy regimens;FOLFOX6 ,FOLFOX6 chemotherapy regimens；ECF，ECF chemotherapy regimens.*

Supplementary File S8.Adverse events reported in the included studies

Study	Adverse events
Wang 2018	*NA*
Ma et al 2020	*NA*
Hao et al 2018	*NA*
Zhu et al 2019	*The erythrocytes, hemoglobin, leukocytes and neutrophils of the TCM group were better than those of the control group, but there were no statistically significant differences in liver and kidney functions between the two groups.*
Li 2020	*NA*
Si et al 2019	*The main adverse reactions during treatment in both groups were bone marrow suppression, gastrointestinal reactions, hand-foot syndrome and peripheral neurotoxicity, but the degree of bone marrow suppression in the TCM group was significantly less than that in the control group. The differences in other adverse effects were not statistically significant.*
Chen et al 2019	*NA*
Wang et al 2016	*The gastrointestinal reactions and bone marrow transplantation were less severe in the TCM group than in the control group.*
Gao 2019	*The incidence of leukopenia, nausea and vomiting, and anorexia in the TCM group was significantly lower than that in the control group.*
Fan et al 2015	*There was no statistically significant response in the GI tract between the TCM and treatment groups.*
Yong et al 2021	*The myelosuppression, neurotoxicity and gastrointestinal reaction in the observation group were less serious than the control group.*
Ai et al 2020	*NA*
Gu et al 2021	*NA*

Supplementary File S9.Data table of HM components, targets and pathways were included.

Component	Degree
quercetin	161
kaempferol	74
Stigmasterol	53
luteolin	48
isorhamnetin	36

Data table of HM components were included

Target	Degree
TP53	117
AKT1	114
CASP3	102
TNF	100
VEGFA	100

Data table of HM targets were included.

Pathway	GO	Count	%	LogP
Pathways in cancer	hsa05200	61	47.28682171	-71.43891209
Hepatitis B	hsa05161	32	24.80620155	-43.95627822
Prostate cancer	hsa05215	26	20.15503876	-39.42753902
Hepatitis C	hsa05160	29	22.48062016	-38.82892583
Pancreatic cancer	hsa05212	24	18.60465116	-38.35228979
Kaposi sarcoma-associated herpesvirus infection	hsa05167	30	23.25581395	-37.71033118
Human cytomegalovirus infection	hsa05163	31	24.03100775	-37.3550231
Bladder cancer	hsa05219	20	15.50387597	-36.66107944
Cellular senescence	hsa04218	26	20.15503876	-33.50293177
Endocrine resistance	hsa01522	23	17.82945736	-33.34108111
Non-small cell lung cancer	hsa05223	21	16.27906977	-32.68970456
Hepatocellular carcinoma	hsa05225	26	20.15503876	-32.61101622
Human T-cell leukemia virus 1 infection	hsa05166	26	20.15503876	-29.31688318
Colorectal cancer	hsa05210	20	15.50387597	-28.89614904
Epstein-Barr virus infection	hsa05169	25	19.37984496	-28.79661502
Platinum drug resistance	hsa01524	19	14.72868217	-28.50078386
Chronic myeloid leukemia	hsa05220	19	14.72868217	-28.12462782
Human papillomavirus infection	hsa05165	28	21.70542636	-27.57862539
Small cell lung cancer	hsa05222	19	14.72868217	-26.3720016
Melanoma	hsa05218	17	13.17829457	-24.70804428

Information of the key pathways of HM included

Supplementary File S10.Proof of English polish

editage

Editing Certificate

This document certifies that the manuscript listed below has been edited to ensure language and grammar accuracy and is error free in these aspects. The edit was performed by professional editors at Editage, a division of Cactus Communications. The author's core research ideas were not altered in any way during the editing process. The quality of the edit has been guaranteed, with the assumption that our suggested changes have been accepted and the text has not been further altered without the knowledge of our editors.

MANUSCRIPT TITLE

Effects of botanical drugs in the Treatment of Cancer-related Fatigue in Patients with Gastric Cancer: a Meta-analysis and Prediction of Potential Pharmacological Mechanisms Using network analysis

AUTHORS

WANG Ziming WU Zihong XIANG Qiong YANG Jingyi XIA Zhenzhong HAO Aohan SONG Enfeng ME Shasha

ISSUED ON

August 02, 2022

JOB CODE

WAZIM_1

THE EDITAGE Promise QUALITY GUARANTEE

Vikas Narang

Vikas Narang
Chief Operating Officer - Editage

editage

Editage, a brand of Cactus Communications, offers professional English language editing and publication support services to authors engaged in over 1300 areas of research. Through its community of experienced editors, which includes doctors, engineers, published scientists, and researchers with peer review experience, Editage has successfully helped authors get published in internationally reputed journals. Authors who work with Editage are guaranteed excellent language quality and timely delivery.

GLOBAL :
+1(833) 979-0061 | request@editage.com

CHINA :
400-120-3020 | fabiao@editage.cn

CACTUS.

IMPACT SCIENCE impact.science

researcher.life

CACTUS lifesciences.cactusglobal.com

editage.com | editage.co.kr | editage.jp | editage.cn | editage.com.br | editage.com.tw | editage.de
